# β-Lactam potentiators to re-sensitize resistant pathogens: Discovery, development, clinical use and the way forward

**DOI:** 10.3389/fmicb.2022.1092556

**Published:** 2023-03-10

**Authors:** Lekshmi Narendrakumar, Medha Chakraborty, Shashi Kumari, Deepjyoti Paul, Bhabatosh Das

**Affiliations:** Functional Genomics Laboratory, Infection and Immunology Division, Translational Health Science and Technology Institute, Faridabad, India

**Keywords:** antibiotic resistance, antibiotic potentiators, β-lactams, β-lactamases, β-lactamase inhibitors, molecular docking, multidrug resistance

## Abstract

β-lactam antibiotics are one of the most widely used and diverse classes of antimicrobial agents for treating both Gram-negative and Gram-positive bacterial infections. The β-lactam antibiotics, which include penicillins, cephalosporins, monobactams and carbapenems, exert their antibacterial activity by inhibiting the bacterial cell wall synthesis and have a global positive impact in treating serious bacterial infections. Today, β-lactam antibiotics are the most frequently prescribed antimicrobial across the globe. However, due to the widespread use and misapplication of β-lactam antibiotics in fields such as human medicine and animal agriculture, resistance to this superlative drug class has emerged in the majority of clinically important bacterial pathogens. This heightened antibiotic resistance prompted researchers to explore novel strategies to restore the activity of β-lactam antibiotics, which led to the discovery of β-lactamase inhibitors (BLIs) and other β-lactam potentiators. Although there are several successful β-lactam-β-lactamase inhibitor combinations in use, the emergence of novel resistance mechanisms and variants of β-lactamases have put the quest of new β-lactam potentiators beyond precedence. This review summarizes the success stories of β-lactamase inhibitors in use, prospective β-lactam potentiators in various phases of clinical trials and the different strategies used to identify novel β-lactam potentiators. Furthermore, this review discusses the various challenges in taking these β-lactam potentiators from bench to bedside and expounds other mechanisms that could be investigated to reduce the global antimicrobial resistance (AMR) burden.

## 1. Introduction

Since the discovery of benzylpenicillin by Sir Alexander Fleming, antibiotics are considered the most important lifesaving drugs and have been nick-named the “magic bullets.” Today, there are different classes of antibiotics used to treat several bacterial infections, and they are classified based on their structure, spectrum, and mode of action (Bush and Jacoby, 2010). Among the different classes of antibiotics, the β-lactam antibiotics are the most widely used and prescribed (Wilke et al., 2005). In the past decade, β-lactams represented more than 60% of all prescriptions (Damlin et al., 2020). This class of antibiotics is characterized by the β-lactam ring in their structure and includes the penems (penicillins), cephems (cephalosporins and cephamycins), carbacephems, carbapenems and monobactams. However, with the growing use of antibiotics, the threat of antimicrobial resistance (AMR) among the bacterial pathogens has also loomed. Due to the antibiotic selection pressure, microorganisms gain AMR mostly by horizontal gene transfer or by accumulating spontaneous mutations in the target genes (Munita and Arias, 2016; Narendrakumar et al., 2020). *Enterococcus* spp., *Staphylococcus aureus*, *Klebsiella pneumoniae*, *Acinetobacter baumannii*, *Pseudomonas aeruginosa*, and *Escherichia coli*, collectively termed the ESKAPE pathogens, are predominantly responsible for infectious diseases in humans and have been identified to be the major contributors to resistance and resistance dissemination (Zhen et al., 2019). Currently, there are increasing reports of difficult to treat (DTR) pathogens resistant to most first-line therapy agents from around the world, and experts have predicted that this growing AMR can possibly cause an “antibiotic apocalypse” where bacterial infections will become no longer treatable by antibiotics.

The World Health Organization (WHO) has stated that AMR is one of the top 10 global public health threats that humanity is facing today (World Health Organization, 2014; Munita and Arias, 2016). The economic cost of AMR is significant, and it has been identified as a major burden for low-and middle-income communities (World Bank, 2017). Considering the impact of AMR in the health sector and the economy, there have been several initiatives to contain the growing AMR burden. This included enforcement of strict rules on the indiscriminate use of antibiotics, surveillance of AMR and AMR drivers (Infectious Diseases Society of America (IDSA), 2011), “One Health” approach to address and avert AMR (Zhen et al., 2019; Mares et al., 2021), extensive research on identifying novel antibiotics and alternatives to antibiotics like vaccines, probiotics, and anti-virulence agents to curb AMR.

The present review expounds one such major strategy, “antibiotic potentiation” with special emphasis to the most used class of antibiotics, the β-lactams. The term antibiotic potentiation refers to the technique by which the activity of an antibiotic is increased, improved or restored. Compounds, either natural or synthetic, that do not exert direct antibacterial property but increase the efficacy of the antibiotic have been used to revive the activity of the antibiotics. Such compounds work either by blocking the primary resistance mechanism of the bacterial pathogen thus rendering the antibiotic effective, or work on the antibiotic to improve its antibacterial activity (Chawla et al., 2022). Several β-lactam potentiators, primarily the β-lactamase inhibitors (BLI) are already in the clinical use. Clinically approved BLIs include clavulanic acid, sulbactam, tazobactam, avibactam, relebactam and vaborbactam. In the present review, the first section recapitulates the various β-lactam antibiotics, their modes of action, and bacterial resistance to them, while the second section summarizes recent developments in β-lactam potentiator discovery, novel potentiators, various strategies used to identify them and the major roadblocks in bringing them from bench to bed-side.

## 2. β-Lactam antibiotics and their mechanism of action

From early 1920s to late 1940s, various research groups had observed antibacterial activity of several species of fungi against different clinically important bacteria. The most common fungi observed to exhibit potential antibacterial activity was the *Penicillium*. Penicillin was isolated from *Penicillium notatum*, but the active compound for clinical trials was produced on a large scale from *Penicillium chrysogenum* (Chain, 1979). Though different *Penicillium* strains produced penicillin, all compounds were identified to have a common four-membered ring with an amide function called the “β-lactam ring.” Penicillin was discovered in 1928 and used clinically by 1940. Later, cephalosporin C was identified and isolated from *Cephalosporium acremonium* in 1945 and the prototype drug for carbapenem was isolated from *Streptomyces* spp. in 1980s (Burton and Abraham, 1951). Monobactams, produced by *Pseudomonas acidophila* and *Pseudomonas mesoacidophila* were discovered more recently (Gwynn et al., 1988). Unlike the 6-aminopenicillanic acid (6-APA) nucleus of the penicillins, cephalosporins have a 7-aminocephalosporinic acid (7-ACA) nucleus while the carbapenems have a five-membered carbon ring associated with a β-lactam ring and monobactams contain a monocyclic ring structure. The β-lactam antibiotics were identified to have a broad spectrum of action and are effective in treating Gram-negative, Gram-positive and anaerobic bacteria. The β-lactam antibiotics have been used to treat infections such as pneumonia, sinusitis, pharyngitis, urinary tract infections (UTIs), skin and soft tissue infections and bloodstream infections (Bush and Bradford, 2016). According to a study published in 2020 by Vincent et al., β-lactam antibiotics were also the most commonly used antibiotics in intensive care units (ICUs), accounting for ~36% of all antibiotics used in treating critically ill patients (Vincent et al., 2020).

The β-lactam antibiotics are bactericidal in nature and the primary mechanism of action of the β-lactam antibiotics is by binding to a group of inner-membrane bound enzymes known as the “penicillin binding proteins (PBPs)” which are important in bacterial peptidoglycan wall synthesis. In Gram-negative bacteria, this peptidoglycan cell wall is ensconced by the bacterial outer membrane (OM), which to an extent hinders the antibiotic penetration (Bush and Macielag, 2010). However β-lactam antibiotics pass through the Gram-negative bacterial porins, reach the peptidoglycan and exert their action (Kim et al., 2020). The mechanism of action of β-lactam antibiotics against Gram negative and Gram positive bacteria is presented in Supplementary Figure 1. The major classes of β-lactam antibiotics, their spectrum of activity, major molecular targets and resistance mechanisms have been summarized in Supplementary Table 1. Structures of major classes of β-lactam antibiotics is presented in Supplementary Figure 2.

### 2.1. Penicillins

Penicillin was the first β-lactam antibiotic discovered and the second antibiotic to be used in human medicine after the sulfonamides. The group contains several natural and semisynthetic compounds and their pharmacokinetics and spectra of action differs significantly. Penicillin G (benzylpenicillin) and Penicillin V (phenoxymethylpenicillin) are the two natural penicillins used in human medicine mostly used to treat Gram-positive bacterial infections (Kulkarni et al., 2019). Among the semisynthetic penicillins, aminopenicillins like ampicillin, developed by the addition of an amino group to the benzylpenicillin, are the most clinically used antibiotics. Amoxicillin, developed by adding hydroxyl group to ampicillin was among the first broad spectrum penicillins that displayed activity against both Gram-positive and Gram-negative bacteria due to its better water solubility and absorbed from gastrointestinal tract. However, emergence of penicillinase producing bacteria prompted researchers to look for novel molecules that resisted penicillinase activity and thus methicillin was developed. Sooner, isoxazolyl penicillins which included the oxacillin, cloxacillin and dicloxacillin also known as antistaphylococcal penicillins, which had better oral absorption than methicillin were introduced to treat infections caused by Penicillin G resistant staphylococci (Smith et al., 1962). In short order, several other semisynthetic penicillins such as carboxypenicillins, acylureidopenicillins and amidino penicillins with improved action and bioavailability were developed, collectively known as the extended spectrum penicillins.

### 2.2. Cephalosporins

The cephalosporin antibiotics have a dihydrothiazine ring fused to the β-lactam nucleus with a 6-membered sulfur containing ring. Cephalosporins are the most frequently prescribed class of β-lactam antibiotics because of their broad spectrum of activity and lower allergenic and toxicity risks (Bush and Bradford, 2016). The use of cephalosporins extensively improved the treatment of infectious diseases and drastically reduced morbidity and mortality rates in pneumonia, meningitis, secondary infections in cancer patients, complicated skin and soft tissue infections, urogenital infections, infective endocarditis, serious bone and joint infections and *Salmonella* infections in children (Kwak et al., 2017; Kapil et al., 2019). Based on the spectra of action, cephalosporin groups of antibiotics have been classified into five generations.

### 2.3. Monobactams

Aztreonam is the most used monobactam antibiotic (Groman, 2009). Monobactam antibiotics are characterized by a single β-lactam ring not fused to another ring like other β-lactams. These antibiotics were identified to be more effective against the Gram-negative bacteria as compared to the Gram-positive bacteria due to their lower affinity to PBPs (Brogden and Heel, 1986). Surprisingly, monobactam antibiotics are resistant to bacterial metallo-β-lactamases (MBL), but susceptible to serine β-lactamases (SBL). Interestingly, Blais et al. had recently reported the discovery of a novel monobactam antibiotic, LYS228 effective against both MBL and SBL producing *Enterobacteriaceae* (Blais et al., 2018). The antibiotic is now in phase II clinical trials in patients with UTI and intra-abdominal infections.

### 2.4. Penems

Penems, the broad spectrum β-lactam antibiotics are majorly synthetic in nature and are sub-grouped as alkylpenems, aminopenems, arylpenems, oxypenems and thiopenems which are distinguished by the side chain of the unsaturated five-membered ring. Carbapenems are the most common penem antibiotic used which differs from other groups by possessing double bond between C2 and C3 and a substitution of carbon for sulfur at C1 (Papp-Wallace et al., 2011). One among the most used carbapenem antibiotics is the amidine derivative of thienamycin, Imipenem (N-formimidoyl-thienamycin) which is a broad spectrum β-lactam antibiotic effective against both Gram-positive and Gram-negative bacteria. However, imipenem is rapidly degraded by kidney dehydropeptidase-I (DHP-I). Hence, another broad spectrum carbapenem antibiotic, meropenem was developed which was relatively stable to DHP-I and that possessed enhanced activity against *Enterobacteriaceae*, *P. aeruginosa, Haemophilus influenzae,* and *Neisseria gonorrhoeae*. Other newly developed carbapenem antibiotics include doripenem, panipenem and biapenem (Perry and Ibbotson, 2002; Goa and Noble, 2003; Greer, 2008).

## 3. Resistance against β-lactam antibiotics: Major threat to the mighty class

It has been estimated that the annual sales of the β-lactam antibiotics is US $15 billion in the international market accounting for 65% of the total antibiotics sold (Thakuria, 2013; Graham et al., 2016). While third and fourth generation cephalosporins, carbapenems and monobactams are the most used β-lactam antibiotics in human medicine, penicillin G is the β-lactam antibiotic used in veterinary medicine. With the increase in use of these antibiotics in various sectors, there has been a surge in resistance acquisition by diverse bacterial species as part of their evolution for survival. Bacteria gain resistance to β-lactam antibiotics mainly by three mechanisms. While production of β-lactamase enzymes (EC 3.5.2.6) serves as the most common cause of resistance against this diverse antibiotic group, loss or alteration of porins and upregulation of efflux pumps are other common mechanisms.

High prevalence of β-lactam antibiotic resistance, especially toward last generation cephalosporins and carbapenem antibiotics has been widely reported in clinically important bacterial pathogens worldwide. In a study conducted by Feretzakis and group in South Eastern Europe, more than 55% of *P. aeruginosa,* one of the major pathogen causing infections in cystic fibrosis (*CF*) patients was identified to be resistant to carbapenem antibiotics, imipenem, meropenem and doripenem and 15% of the isolates were resistant to cephalosporin antibiotics, cefepime and ceftazidime (Feretzakis et al., 2019). *Pseudomonas aeruginosa* is also known to cause infection in patients with chronic obstructive pulmonary disease (COPD), diabetes mellitus and also cause nosocomial infections. Another major pathogen imparting high AMR burden is the *K. pneumoniae* causing sepsis, pneumonia, surgical site infections and UTIs (Fursova et al., 2021). About 60% of *K. pneumoniae* isolated from India were reported to be resistant to carbapenem antibiotics (OECD and World Health Organization, 2020). Emergence and spread of carbapenem resistant *A. baumanii* has caused significant alarm in recent times. *A. baumanii* is associated with many nosocomial infections including ventilator associated pneumonia (VAP), bloodstream infections, UTI and wound infections (Necati Hakyemez et al., 2013). A plethora of β-lactam resistance determinants were identified in these resistant pathogens including genes coding for various β-lactamase enzymes and efflux pump proteins (Necati Hakyemez et al., 2013). Many of these resistance determinants are transferred among the bacteria through horizontal gene transfer and spread to inter and intra bacterial species (Hull et al., 2021).

Additionally, high prevalence of β-lactam heterogenous drug resistance or heteroresistance has been observed in several clinically important pathogens such as *K. pneumoniae* and Methicillin resistant *S. aureus* (MRSA). Heteroresistance is defined as the existence of two different subpopulation of the same microorganism within a single population with difference in resistance profiles (susceptible and resistant) against a common antibiotic. Majority of the Methicillin resistant *Staphylococcus aureus* (MRSA) cultures are identified to have a phenotypically heterogenous population of *S. aureus* strains with one subpopulation with extremely high β-lactam minimum inhibitory concentration (MIC) values and another subpopulation which exhibit very poor resistance (Kim et al., 2013). Similarly, heterogenous drug resistance was observed in MRSA against oxacillin which on single induction with the antibiotic converted into a homogenously resistant population (Chung et al., 2016). Nodari and his team reported high prevalence of imipenem heteroresistance among carbapenemase-producing *Enterobacteriaceae* (CPE; Nodari et al., 2015). Heterogenous drug resistance among bacterial populations are often under-detected and have significant clinical implications as it is one of the major reasons for treatment failure.

### 3.1. β-Lactamase enzymes

β-Lactamases are a diverse group of enzymes that have the ability to hydrolyze the amide bond of the β-lactam ring rendering the antibiotic ineffective. β-Lactamase producing *E. coli* was first identified in 1940’s even before the first β-lactam antibiotic was introduced for clinical use. Currently, there are numerous β-lactamases identified that have narrow or broad-spectrum activity against different β-lactam antibiotics. The sequence information of 7,537 β-lactamases, 1,526 structural information, and kinetics of 47 β-lactamases evolved over the years have been listed in the Beta-Lactamase DataBase (BLDB) revealing the diverse nature of the enzymes (Naas et al., 2017). These enzymes are usually located in the periplasmic space of the bacteria and they hydrolyse the β-lactam antibiotic before they reach the target molecule. Many Gram-negative bacteria also produce membrane vesicles that bear high levels of these enzymes that act on the antibiotics and reduce the concentration even before it reaches the infection foci (Ciofu et al., 2000).

Various characteristics like molecular structure, substrate profile, biochemical properties and functional characteristics were initially used to classify the β-lactamase enzymes. The Amber classification classifies the enzymes into four different classes (class A, B, C, and D) based on its sequence motifs and hydrolytic properties. Class A, C, and D constitute enzymes that employ their serine residue for the nucleophilic attack and hydrolyze of the β-lactam antibiotic while class B enzymes utilize a metal-activated water nucleophile to drive the hydrolytic reaction. There have been various reviews that summarize the different classes of β-lactamases and their substrates (Page, 2002; Nagshetty et al., 2021; Akhtar et al., 2022). Some β-lactamases have been identified to be specific to certain species of bacteria and some have been identified to be successful in global dissemination and confer selective fitness to bacterial clones (Fernández et al., 2012). Many ESKAPE pathogens have been identified to possess more than one class of β-lactamase resistance gene rendering them multidrug resistant and hard to treat (Oliveira et al., 2020). In 1980s, *Enterobacteriaceae* isolates capable of hydrolysing extended spectrum β-lactam antibiotics were reported from around the world and were identified to possess extended spectrum β-lactamases (ESBLs) such as TEM, SHV, and CTX-M. Carbapenem antibiotics were the treatment options for such ESBL producing isolates. However, bacterial isolates resistant to carbapenem antibiotics soon evolved capable of producing carbapenemase enzymes. Genes that confer ESBL activity (*bla*_TEM_*, bla*_SHV_*, bla*_CTX-M_) and carbapenamase activity (*bla*_KPC_) in the class A, carbapenamases (*bla*_NDM_*, bla*_VIM_ and *bla*_IMP_) in the class B, ESBLs (*bla*_CMY_*, bla*_AMP_ and *bla*_ADC_) in class C and the carbapenemase encoding *bla*_OXA_ genes in class D have been explicitly been successful in transmission among the *Enterobacteriaceae* family, *A. baumannii* and *P. aeruginosa* (Raible et al., 2017; Ahmed et al., 2020).

These classes of resistance genes are either chromosomally encoded or plasmid encoded. Plasmid encoded resistance genes have demonstrated significant success in the global spread of AMR among bacteria in clinical and non-clinical settings. KPC encoded by *bla*_KPC_ gene has been identified to have the potential for inter-species and geographical dissemination due to its association with Tn-3 type transposon Tn4401 capable of inserting into different types of plasmids of Gram-negative bacteria (Arnold et al., 2011). Additionally, the global success of KPC dissemination is also attributed to the clonal expansion of *K. pneumoniae* ST258, ST512, ST304, and ST11 (Munoz-Price et al., 2013). Mostly, *bla*_NDM_ variants are plasmid encoded and are rapidly transferred between enterobacterial isolates *K. pneumoniae* and *E. coli.* NDM encoding isolates are resistant to almost all β-lactam antibiotics with tigecycline and colistin being the only treatment options (Wu et al., 2019). Recently, there have been various reports of chromosomal integration of usually plasmid encoded β-lactamase resistance genes like *bla*_CTX-M_ and *bla*_OXA-48_ in *E. coli* isolates (Rodríguez et al., 2014; Turton et al., 2016). In many other cases, co-existence of more than one class of β-lactamase resistance gene in both chromosome and plasmid was observed in Gram-negative bacterial pathogens (Poirel et al., 2010). The β-lactamase resistance genes have been found associated with various mobile genetic elements (MGEs) like plasmids, transposons, insertion sequences (IS) and integrons. *bla*_IMP-1_ has been identified to be associated with Class I integrons which may have chromosomal or plasmid location in different species (Watanabe et al., 1991; Arakawa et al., 1995). Verona integron-encoded metallo-β-lactamase (VIM) is another prevalent integron associated MBL followed by German imipenemase (GIM-1) and Seoul imipenemase (SIM-1) located in class 1 integrons of *Pseudomonas* spp. Insertion sequences like IS*Aba1, ISAba4* and transposons such as Tn*2006 and* Tn*2008* are found associated with the *bla*_OXA-23_ gene in *A. baumannii* chromosome while IS*Aba3* and IS*18* are found associated with *bla*_OXA-58_ (Poirel and Nordmann, 2006; Corvec et al., 2007). Recently Chen et al., reported the identification of a novel ESBL encoded by the gene *bla*_OXA-830_ in the chromosome of environmental bacteria *Aeromonas simiae* (Chen et al., 2019). The OXA-48 like carbapenemases in *Enterobacterales* has been found to be associated with various transposable elements and insertion sequences. *Klebsiella pneumoniae* and *E. coli* circulating in the North Africa and Middle East have been reported to possess OXA-48 associated with Tn*1999* variants on IncL plasmids (Giani et al., 2012). OXA-181 and OXA-232 endemic to the Indian subcontinent and few sub-Saharan African countries are associated with IS*Ecp1*, Tn*2013* on ColE2, and IncX3 types of plasmids. Additionally, clonal dissemination of certain high risk clones of *K. pneumoniae* (ST147, ST307, ST15, and ST14) and *E. coli* (ST38 and ST410) are also a minor cause of the spread of OXA-48 like carbapenemases (Pitout et al., 2019). Many ESBL genes such as *bla*_TEM_*, bla*_SHV_*, bla*_CTX-M_, carbapenemase genes such as *bla*_SPM_*, bla*_IMP_*, bla*_VIM_*, bla*_NDM_, *bla*_KPC_ and *bla*_OXA-48_ are conjugative plasmid encoded in ESKAPE pathogens which allows in the easy transmission of these genes among the bacterial isolates (Kim et al., 2011; Veeraraghavan et al., 2019). Thus, β-lactamase enzymes form the most common method of β-lactam resistance in clinical pathogens.

### 3.2. PBP modification

The PBPs are the target of the β-lactam antibiotics. The PBPs are transpeptidases or carboxypeptidases involved in peptidoglycan metabolism. Hence PBP modification serves as another major mechanism by which bacteria resist the action of the antibiotic. The mutational alterations in PBPs can confer resistance to β-lactam antibiotics (Chesnel et al., 2002). Unemo et al., in 2012 had reported the isolation of a high level cefixime and ceftriaxone resistant *Neisseria gonorrhoeae* having a *penA* mosaic allele with an additional A501P mutation in the PBP2 (Unemo et al., 2012). Moya et al. had previously reported an unusual mechanism of β-lactam resistance by mutational inactivation of non-essential PBPs (PBP4) coded by *dacB* which behaves as a trap target for β-lactams (Moya et al., 2009). Additionally, the mutation on *dacB* also triggers over-expression of AmpC coding β-lactamase enzymes and activating the CreBC (BlrAB) two-component regulator which together results in high level β-lactam resistance. Mutation in the PBP coding gene down regulates its expression which in turn reduces the peptidoglycan turnover. The reduced peptidoglycan synthesis is considered as a cue for over production of β-lactamase enzymes. Recently, Lazzaro et al. reported high-level resistance of *Enterococcus faecalis* to Ceftobiprole (BPR), a new-generation cephalosporin as a result of alterations in the *pbp4* gene (Lazzaro et al., 2022). The mutations revealed to over-express PBP4 leading to increased transpeptidation causing higher peptidoglycan synthesis that prevented Ceftobiprole penetration into the bacterial cell. Thus, mutations leading to gain of function or loss of function of PBP have been determined to play a major role in β-lactam resistance in bacteria.

### 3.3. Selective permeability by porin regulation

Gram-negative bacteria possess the OM that acts as an additional barrier for the transport of solutes into the cell. The selective transport of molecules is achieved by specialized proteins called porins present on the OM. Modulation of porin expression is another mechanism of β-lactam resistance particularly in Gram-negative bacteria. Various insertional elements and point mutations in the promoter and protein coding sequences have been identified to alter porin protein expression and structure. *Escherichia coli* mutants lacking OmpC and OmpF proteins were documented to demonstrate high level resistance to cefoxitin and other cephalosporin antibiotics (Jaffe et al., 1982). Similarly, characterization of meropenem resistant *Serratia marcescens* isolates revealed insertional inactivation of *ompF* gene by an IS element, IS*1* (Suh et al., 2010). Mutations in *ompK*35 and *ompK*36 in *K. pneumoniae* and *ompC* and *ompF* in *Enterobacter* spp. have been associated with increased resistance to the majority of carbapenem antibiotics (Doumith et al., 2009). Pagel et al., noticed that mutations at the L3 loop of OmpU in the cholera causing pathogen, *Vibrio cholerae* resulted in decreased susceptibility of the bacteria to cephalosporin antibiotics (Pagel et al., 2007). Likewise, several studies utilizing either site directed mutagenesis, knockdown or knockout of porin encoding genes in several clinically important pathogens revealed that these pathogens could gain reduced susceptibility to even last generation cephalosporins and carbapenems (Smani et al., 2014; Choi and Lee, 2019). A recent study by Khalifa et al. revealed that 93.3% of *E*. *coli* and 95.7% of *K*. *pneumoniae* isolated from hospitals in Egypt were identified to have lost their porins or showed modified porins resulting in heightened resistance to all carbapenem antibiotics (Khalifa et al., 2021). Interestingly, there have also been reports of association of porin regulation and expression of efflux pumps. Carbapenem resistance in *P. aeruginosa* has been primarily due to a combination of decreased transcriptional expression of OprD and upregulation of MexAB-OprM active efflux system (Muderris et al., 2018). Thus, in clinical isolates, interrelation of different mechanisms of resistance has been identified and mutations in some genes have been identified to activate multiple other resistance pathways.

### 3.4. Efflux pumps

Decreasing passive influx and increasing active efflux of antibiotics is a common mechanism of antibiotic resistance in bacteria. In clinically important pathogens, especially Gram-negative bacteria, a large number of promiscuous/polyselective efflux pumps such as the Resistance-Nodulation-Division (RND) efflux pumps that can recognize and expel a large variety of antibiotics have been identified. Unlike the β-lactamase coding genes that are mostly acquired, genes coding for efflux pumps are mostly intrinsic and found in both susceptible as well as resistant clones. However, acquisition of mutations at the regulatory region, promoter or open reading frame (ORF) of the gene leads to over-expression of these genes that cause resistance. Though the role of efflux pumps in β-lactam drug resistance was widely described in *P. aeruginosa,* it was not until recently that its role was identified in the members of the *Enterobacteriaceae* family (Glen and Lamont, 2021). About 12 different RND efflux pumps have been identified in *P. aeruginosa* of which MexAB-OprM, MexXY-OprM, and MexCD-OprJ play important role in β-lactam resistance and at least one of these efflux pumps have revealed increased expression during antibiotic treatment (Masuda et al., 2000). RND efflux pumps have also been reported in *K. pneumoniae* conferring β-lactam drug resistance. KexD expression was identified to be high in imipenem resistant *K. pneumoniae* and KpnEF (SMR type efflux pump) over-expression was noticed in cephalosporin resistant isolates (Srinivasan and Rajamohan, 2013). Uddin and Ahn recently reported multiple efflux pumps (AcrAB, AcrEF, EmrAB, MdfA, and MdtK) involved in conferring reduced susceptibility of β-lactam antibiotics in *Salmonella* Typhimurium (Uddin and Ahn, 2018). The expression of efflux pumps has been identified to be highly increased in bacterial pathogens during antibiotic treatment thereby significantly increasing the MIC of the drug required for the treatment. The treatment of *Klebsiella aerogenes* with imipenem was identified to drastically increase the expression of the *marA* gene that codes for an efflux pump protein (Bornet et al., 2003). Many other efflux pumps that cause co-resistance to other antibiotics have been identified to co-spread with β-lactam resistance genes (Li et al., 2019). The OqxAB, a multi-drug efflux pump widely attributed to resistance against quinolones, nitrofurantoin and chloramphenicol is co-transferable with *bla*_CTX-M_, *rmtB* and *aac(6′)*-1b etc. The *oqxAB* genes are located either on the chromosome and/or on plasmids and are flanked by IS26-like element in clinical isolates of *Enterobacteriaceae* which makes them transferable (Li et al., 2019). Efflux pumps that expel out multiple antibiotics thus posing threat of multidrug resistance (MDR) in bacteria have been a major concern to both researchers and clinicians alike.

## 4. Strategies to improve efficacy of existing antibiotics

**Figure 1:**
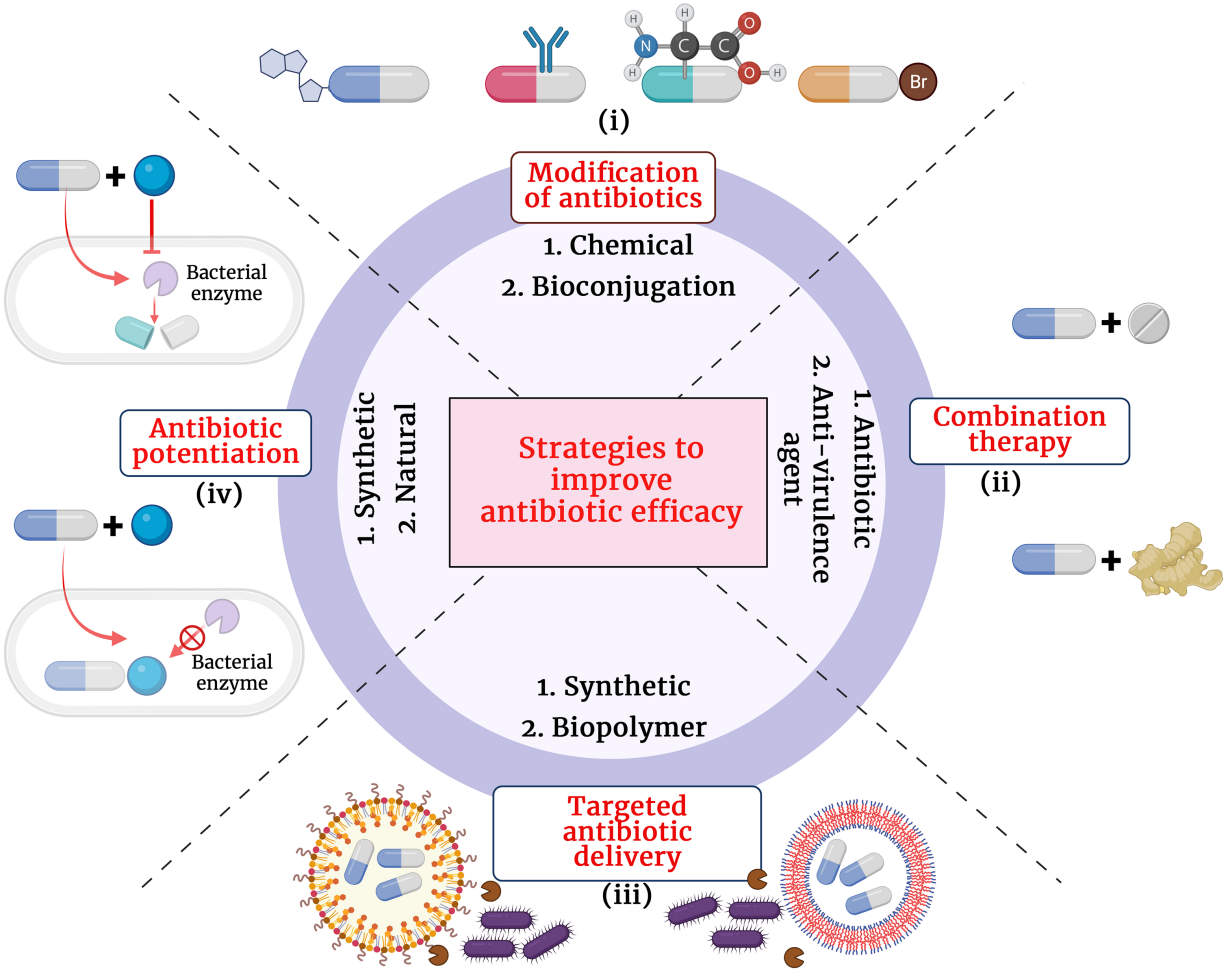
Different strategies adopted to improve the antibiotic treatment efficacy. (i) Modification of antibiotics by chemical and biological methods, (ii) Combination therapy with another antibiotic or anti-virulence agent, (iii) Targeted antibiotic therapy by conjugation with synthetic or biopolymer and (iv) Antibiotic potentiation with synthetic or natural compounds.

The growing antibiotic resistance and the discovery void of novel classes of antibiotics have caused immense pressure and challenge to clinicians and researchers across the globe. Since 1987, for over 30 years, very few new antibiotics with potential antibacterial activity have reached the final phases of clinical trials and have made their way to the market. Though retapamulin, the topically used pleuromutilin antibacterial, was introduced into the market in 2007, the chemical class was first described in 1952 (Novak and Shlaes, 2010). Though there exists a discovery void of novel classes of antibacterial compounds, the antibacterial pipeline has not been empty during the past decades and there have been many antibacterial compounds with improved activity being introduced for trials. The different strategies adopted to improve antibiotic treatment efficacy at the wake of dissemination of antibiotic resistance pathogens are presented in Figure 1.

**Figure 2:**
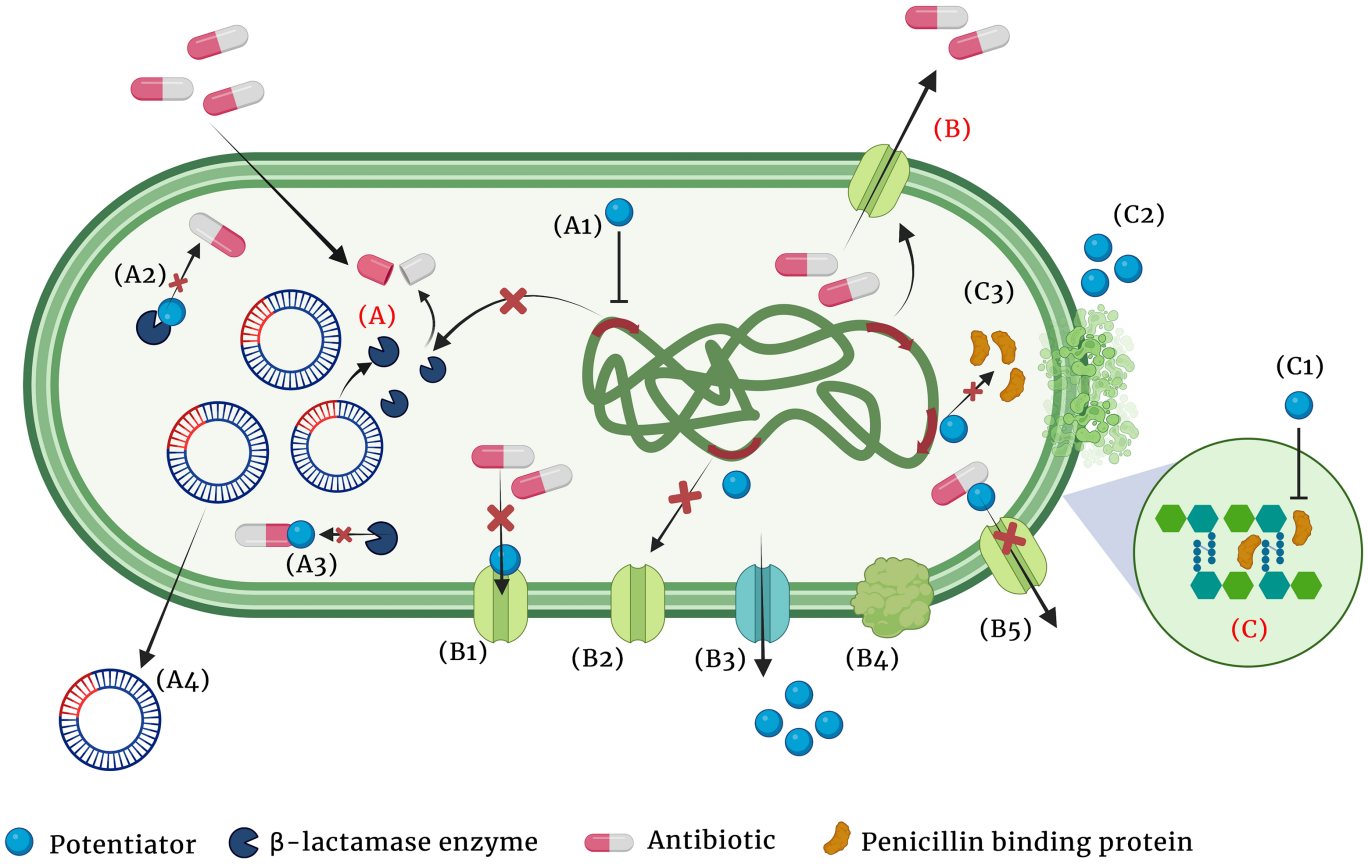
Major mechanisms of β-lactam resistance **(A-C)** and the different means by which the adjuvant potentiates the antibiotic (A1-A4, B1-B5 and C1, C2). **(A)** Resistance by β-lactamase enzyme hydrolysis of the antibiotic, **(B)** Resistance by active efflux of the antibiotic, **(C)** Resistance by PBP modification and increased peptidoglycan synthesis. (A1) Suppression of the enzyme expression by the potentiator, (A2) Modification of the enzyme and inhibiting its activity, (A3) Modification of the antibiotic and making it inaccessible for the enzyme, (A4) Cure the plasmid that encodes the resistance gene, (B1) Block efflux pump and prevent antibiotic efflux, (B2) Suppress expression of efflux pumps, (B3) Competitive inhibition of the antibiotic efflux, (B4) Inhibition of proper efflux pump assembly, (B5) Alter antibiotic structure and prevent its efflux, (C1) Modification of PBP and decrease peptidoglycan synthesis, (C2) Chelation of divalent cations and (C3) Suppression of PBP synthesis.

- Structural modification of antibiotics: Remarkable improvement in the antibacterial activity and stability of the β-lactam antibiotics have been achieved by structural modifications. A modification in the dihydrothiazine ring fused to the β-lactam ring in the cephalosporin nucleus was identified to improve cephalosporin activity against *S. aureus* and *P. aeruginosa* isolates (Neu, 1985). Similarly, introduction of acyl side chains to β-lactam antibiotics ameliorated its antibacterial activity by increasing its affinity toward PBPs (Neu, 1985). Additionally, chemical modifications of β-lactam antibiotics have also been identified to impart multifaceted properties to the class such as antifungal, anti-parasitic and anticancer (Neu, 1985; Hamilton-Miller, 1999). Novel modifications of β-lactam antibiotics with different pharmacophophoric groups and their biological activities were recently reviewed by De Rosa et al. (2021).
- Combination therapy: Combinations of drugs that have different modes of action or targets in the bacterial cell are used to combat antibiotic resistance. Antibiotic combinations that impart synergistic activities have been identified to possess broad spectrum antibacterial activity and less resistance evolution of bacterial pathogens. Combination therapy of β-lactam antibiotics with aminoglycoside antibiotics have revealed improved activity against MDR *P. aeruginosa*, *S. aureus*, *Enterococcus* sp. and *Streptococcus* sp. (Paul et al., 2006). β-lactam antibiotics exert its antibacterial action by inhibiting the bacterial cell wall synthesis while the aminoglycoside antibiotics inhibit the bacterial growth by interfering with the protein synthesis by binding to the aminoacyl site of 16S ribosomal RNA of the 30S ribosomal subunit. Conventionally, combination therapy with a β-lactam antibiotic and aminoglycoside works due to the cell damage caused by β-lactam antibiotic that allow aminoglycoside to penetrate more efficiently into the bacterial cell and exert its action. However, the exact detailed mechanism of the synergism is not clear. Also, recent reports revealed enhanced synergistic activity of carbapenem antibiotics with peptide antibiotic daptomycin (Sakoulas et al., 2017). Daptomycin is a cyclin anionic lipopeptide which is usually ineffective against Gram negative pathogens due to the presence of its OM. However, daptomycin along with β-lactam antibiotics show synergism as the peptidoglycan damage caused by β-lactam antibiotic could help daptomycin to enter the bacterial cell and cause membrane depolarization and also inhibit DNA, RNA and protein synthesis. However, the exact molecular bases and mechanism of action involved in carbapenem antibiotic and daptomycin interaction remain unknown (Gagetti et al., 2016). Siriyong et al., had recently reported the effectiveness of dual β-lactam antibiotic therapy to be effective against MDR *P.aeruginosa* (Siriyong et al., 2019). This activity is due to the double dose effect. Combining two β-lactam antibiotics enables inactivation of multiple PBPs to achieve synergistic bacterial killing. Nevertheless, clinical data to support combination therapy of β-lactam antibiotics with other classes of antibiotics is sparse and recent reports suggested that dual β-lactam or carbapenem therapy was not superior to single carbapenem regimens (Rigatto et al., 2022). Apart from antibiotic-antibiotic combination therapies, combining an antibiotic with an anti-virulence compound has also been identified to be an efficient way to combat MDR pathogens with minimal resistance progression (Rezzoagli et al., 2020). Anti-virulence compounds are those that target the pathogen’s virulence pathways rather than pathways for its survival. Cefiderocol that combines a catechol type siderophore and cephalosporin core with side chains similar to cefepime and ceftazidime has been identified to be effective against Gram-negative pathogens, including carbapenem-resistant *Enterobacterales* (Wu et al., 2020). The combined structure of siderophore-cephalosporin confer enhanced stability against hydrolysis by ESBL, CTX-M, and carbapenemases, such as KPC, NDM, VIM, IMP, OXA-23, OXA-51-like and OXA-58. Moreover, the siderophore that chelate extracellular iron enhances the penetration of the molecule into the bacterial cells through the iron transporter channels in addition to the passive diffusion through membrane porins (Wu et al., 2020).
- Targeted antibiotic delivery: Apart from the above strategies, another method to increase the efficacy of antibiotics is to achieve targeted and durable drug delivery. Targeted drug delivery increases the amount of antibiotic at the infection foci and increased durability allows more time for the drug to act upon the bacteria. Complete killing by the antibiotics prevents bacterial evolution to attain resistance. Nanoscale delivery systems which are either biological lipid based, or synthetic polymer based are popularly used to deliver the antibiotic to the site of infection (Stebbins et al., 2014). These delivery systems protect the antibiotic from enzymatic degradation by antibiotic modifying enzymes and additionally, antibody conjugated delivery systems specifically target the pathogenic bacteria and also activate host immunity at the site of infection thereby enhancing pathogen killing (Abed et al., 2015).
- Antibiotic potentiators: Another strategy used to battle the growing antibiotic resistance is to preserve the activity of existing antibiotics with non-antibiotic compounds that could restore their activity. Such compounds that do not exert direct antibacterial property, but increase the efficacy of the antibiotic or restore the activity of the drug are collectively known as antibiotic potentiators, adjuvants or resistance breakers (Laws et al., 2019). Figure 2 depicts the various mechanisms by which bacteria resist antibiotics and how potentiators function. In addition to not having antibacterial activity, it is important that the antibiotic potentiators should have other characteristics such as ideal pharmacological features when administered along with the antibiotic, high serum stability, high bioavailability, and low toxicity. The potentiators should also ideally be highly selective and have no eukaryotic targets. Additionally, it is ideal that the potentiator compounds be easily available and economical.

## 5. β-Lactam potentiators

β-lactam potentiators are mainly of three types, *viz.* (i) β-lactamase inhibitors (BLIs), (ii) efflux pump inhibitors (EPI), and (iii) membrane permeabilizers. Among the three, the BLIs are the most successful and widely used in the clinical setting. There are several natural and synthetic β-lactam potentiators developed. In the context of rising AMR, many non-conventional potentiators are also being studied to evaluate their potency in clinical settings.

### 5.1. β-Lactamase inhibitors – success stories and clinical status

The BLIs are compounds that render the β-lactamase enzymes produced by the bacterial isolates inactive to hydrolyze the antibiotic. The emergence of β-lactamase producing isolates were reported from clinical settings soon after the introduction of β-lactam antibiotics. Though several macromolecular fractions (MM4550, MM13902, MM17880, BRL1437) from different *Streptomyces* sp. were identified to pose β-lactamase inhibitory activity, they were not clinically translated due to their unstable nature and undesirable pharmacological profiles. Reading and Cole in the late 1970s reported the detection of a compound with novel clavum structure isolated from *Streptomyces clavuligerus* ATCC 27064 (Reading and Cole, 1977). The compound when used in combination with β-lactam antibiotics significantly enhanced its antibacterial activity against penicillinase and cephalosporinase producing *E. coli*, *K. aerogenes*, *Proteus mirabilis*, and *S. aureus* isolates. This compound identified to be clavulanic acid was the first clinically potent BLI. Soon, a combination of amoxicillin and clavulanic acid (Amoxiclauv/Augmentin) was used clinically and found highly successful against class A β-lactamase producing bacterial isolates. The similarity of stereochemistry between clavulanic acid and β-lactamase enzymes was identified as the major contributing factor for its potentiation activity (Reading and Cole, 1977). Some BLIs are by themselves β-lactam antibiotics that at sub-inhibitory concentration augment the activity of the partner β-lactam antibiotic.

Following the identification of clavulanic acid, the β-lactamase inhibitory activities of various synthetic penicillin-based sulfones were screened which led to the discovery of sulbactam and tazobactam (YTR 830; Akova, 2008). Sulbactam, the halogenated derivatives of penicillanic acid (6-β-bromo-and 6-β-iodopenicillanic acid), was identified to be effective against class A β-lactamase producing Gram-negative isolates (Rolinson, 1991). However, it was identified to be less potent as compared to clavulanic acid in its activity against *S. aureus* β-lactamases and bacteria that possess TEM variants. While sulbactam was combined with ampicillin, amoxicillin, piperacillin, cefoperazone, cefotaxime and cefepime, tazobactam is used in combination with piperacillin and ceftolozane. The piperacillin-tazobactam combination was identified to be effective against broad-spectrum β-lactamases-producing and some extended-spectrum β-lactamases-producing *Enterobacteriaceae*. However, the combination was not effective against AmpC β-lactamases producing Gram-negative bacteria (Rolinson, 1991; Gin et al., 2007). Ceftolozane-tazobactam combination was identified to be even effective against Pseudomonas. As a therapeutic value addition, recent preliminary *in vitro* studies by Sakoulas et al., revealed that tazobactam potentiate peptide antibiotic, daptomycin against MRSA and colistin resistant *A. baumanii* (Sakoulas et al., 2017). An interesting finding by Urban et al. revealed that different concentrations of the BLIs have different targets. Clavulanic acid, sulbactam and tazobactam at lower concentration were identified to manifest the potentiation activity by inhibiting the β-lactamase activity, while at higher concentration, it exerted its potentiation activity through PBP modulation (Urban et al., 1995). Important BLIs, their class, inhibition spectrum and clinical status are listed in Table 1.

**Table 1:** List of important BLIs, their class, inhibition spectrum, and clinical status.

Class of β-lactamase inhibitor	Inhibitor	Partner β-lactams	Inhibition spectrum	Current status	Isolated from	References
First generation						
Irreversible acylation of catalytic serine residue	Clavulanic acid	Amoxicillin, Ticarcillin	Plasmid encoded Class A ESBLs	Completed, marketed as Augmentin^®^, Timentin^®^	*Streptomyces clavuligerus*	Bush (1988)
Penicillin-based sulfones	Sulbactam	Ampicillin, Piperacillin, Amoxicillin, Cefoperazone	Primarily to Class A (TEM-type β-lactamases), less effective against SHV and OXA-type β-lactamases	Completed, marketed as Unasyn^®^	Synthetic	Benson and Nahata (1988)
	Tazobactam (YTR 830)	Piperacillin, Ceftolozane	Class A ESBLs (TEM, SHV), Class C enzymes and OXA-type β lactamases	Completed, marketed as Zosyn^®^	Synthetic derivative of penicillin	Wise et al. (1991) and Bush (2015)
Second generation						
Diazabicyclooctane (DABCO)	Avibactam (AVE1330A, NZL 104)	Ceftaroline, Ceftazidime, Aztreonam	Class A ESBLs (TEM, KPC and SHV) Class A carbapenemases, Class C and D enzymes,	Completed, marketed as Avycaz^®^, Zavicefta^®^	Synthetic	Bonnefoy (2004) and Ehmann et al. (2012)
	Relebactam (MK-7655)	Imipenem	Class A ESBLs, Class A carbapenemases Class C enzymes	Completed, marketed as Recarbrio^®^	Synthetic	Blizzard et al. (2014) and FDA (2019)
Third generation						
Boronic acid inhibitor	Vaborbactam	Biapenem, Meropenem, Doripenem, Ertapenem	Broad spectrum of class A (KPC, CTX-M, SHV), C (CMY) and D enzymes	Completed phase III trials, marketed as Vabomere™, Carbavance^®^	Synthetic	Hecker et al. (2015)
	(VNRX-5133)	Cefepime	Class A, C and D serine β lactamases and class B MBLs (VIM and NDM) in both carbapenem-resistant Enterobacteriaceae and *P. aeruginosa*	Undergoing phase III drug–drug interaction study	Synthetic	Liu et al. (2020)
Bicyclo-acyl hydrazide	Zidebactam (WCK 5107)	Cefepime	AmpC, ESBL, KPC, and OXA-48-like β-lactamases	Under phase III clinical trials	Synthetic	Yahav et al. (2020) and Karlowsky et al. (2020)
Diazabicyclooctane	Nacubactam (OP0595, RO7079901, RG6080)	Meropenem	Class A, Class C and some Class D β-lactamases and against carbapenem-resistant *K. pneumoniae*	Phase I clinical trial completed	Synthetic	Mallalieu et al. (2020)
Diazabicyclooctane	Durlobactam (ETX2514)	Sulbactam	Class A, C and D serine β-lactamases.	Phase III clinical trial completed	Synthetic	Shapiro et al. (2021)
Diazabicyclooctane	ETX1317/ Prodrug ETX0282	Cefpodoxime proxetil	Class A, C, and D serine β-lactamases	Phase I clinical trial completed	Synthetic	Durand-Réville et al. (2020)
Penicillanic acid sulfone	Enmetazobactam (AAI101)	Cefepime	CTX-M, TEM, SHV, and other class A β-lactamases	Phase III clinical trial completed	Synthetic	Papp-Wallace et al. (2019)
Metallo-beta-lactamases (MBL) inhibitors	Elores (CSE:1034)	Ceftriaxone, sulbactam, disodium edetate (EDTA)	MBLs	Completed, marketed in India as Elores	EDTA	Chaudhary et al. (2017)
	ANT 431	Meropenem	MBLs and MBL-producing carbapenem resistant *Enterobacteriaceae* (CRE)	Undergoing clinical trials	Synthetic	Everett et al. (2018)
	QPX7728	Meropenem	VIM-1, NDM-1 and IMP-1	Under phase I clinical trials	Synthetic	Lomovskaya et al. (2021)
	Magnolol	Meropenem	NDM	Undergoing clinical trials	*Magnolia officinalis*	Liu et al. (2018)
	Aspergillomarasmine A	Meropenem	NDM-1 enzyme and other MBLs, VIM-2	Undergoing clinical trials	*Aspergillus versicolor*	King et al. (2014)
	Bisthiazolidines	Meropenem	Class B1,B2 and B3 MBLs	Undergoing laboratory studies	Synthetic	Hinchliffe et al. (2016)
	Nitrilotriacetic acid and *N*-(phosphonomethyl)iminodiacetic acid	Meropenem	VIM, NDM and IMP classes		6-phosphonomethylpyridine-2-carboxylates	Tehrani et al. (2020)

Recently, two new BLIs of the diazabicyclooctanones (DBOs) group namely avibactam and relebactam were identified with enhanced carbapenamase inhibitory activity. They were the first non β-lactam BLI to be used clinically and they exerted their β-lactamase inhibitory activity by acylating the β-lactamases reveribily unlike clavulanic acid, sulbactam or tazobactam that exerted irreversible inactivation of the resistance enzyme (Ehmann et al., 2012). Avibactam potentiated ceftaroline, ceftazidime, aztreonam while relebactam was used in combination with imipenem (Yahav et al., 2020). Interestingly, relebactam at the same concentration potentiates imipenem by its β-lactamase inhibitory activity and also PBP inhibitory activity (Blizzard et al., 2014). Successive to DBO BLIs, several boronic acid based BLIs were developed which were found to be effective against β-lactamase producing bacteria and they are at different stages of clinical trials. Most of the boronic acid based BLIs were identified to have dual mode of action like the relebactam. They exert their potentiation activity by β-lactamase inhibition and also PBP modification thereby enhancing their spectrum. Boronic acid based BLIs were active against most of the Class A, C and D (Oxa-48) β-lactamases (Lepak et al., 2019; Liu et al., 2020). Some boronic acid based BLIs such as zidebactam (WCK 5222) and taniborbactam (VNRX-5133) were active against Gram-negative organisms producing various carbapenemases, including MBLs producers (Lepak et al., 2019; Liu et al., 2020).

Though there are various BLIs developed against SBL (Amber classes A, C and D), effective MBL inhibitors are sparse. Despite a large amount of research being carried out to identify a potent MBL inhibitor, very few have reached the clinical trials and none have been commercialized. Elores (CSE-1034: ceftriaxone+sulbactam+disodium edetate) is the only MBL inhibitor used in patients with ESBL/MBL producing bacterial infections. The drug is approved in India by the Indian FDA after phase III clinical trials (Chaudhary et al., 2017). The major mechanism by which MBL inhibitors exert their activity is metal chelation. Elores showed high treatment success (83%) against both ESBL as well as MBL producing pathogens and lower rate of adverse effects (8.4%) caused by other drugs of choice to treat the infections (Chaudhary et al., 2017). However, long term usage of sodium edetate is not advisable as the chelating agent could interfere with eukaryotic enzymes that utilizes divalent cations for their activity. Aspergillomarasmine A, a natural compound discovered to have inhibitory activity against NDM-1 and VIM-2 exert its action by chelating the zinc ion present at the enzyme’s active site (King et al., 2014). Bisthiazolidines and small bicyclic compounds also utilize the same mechanism and were found to be effective against B1, B2, and B3 MBLs (Hinchliffe et al., 2016). Currently, development of covalent inhibitors that can bind to specific conserved amino acids at the active site of MBLs are gaining momentum. Irreversible inhibition of MBL (IMP-1) by 3-(3-mercaptopropionylsulfanyl) propionic acid pentafluorophenyl ester by binding to Lys224 and activity of ebselen against NDM-1 by binding to Cys221 are examples for such strategies (Kurosaki et al., 2005; Chiou et al., 2015). The major MBL inhibitors have been recently reviewed by Li et al. (2022). Ceftazidime-avibactam, ceftolozane-tazobactam, ceftazidime-tazobactam, meropenem-vaborbactam and imipenem-relebactam are the recently developed β-lactam-BLI combinations used to treat ESBL and carbapenemase producing bacterial pathogens (Yahav et al., 2020). However, very limited BLIs have been identified that pose both SBL and MBL inhibitory activity and the quest for such compounds continues.

### 5.2. β-Lactam efflux pump inhibitors

Efflux pumps are a mechanism by which bacteria counteract the action of antibiotics by pumping out the drug thereby significantly decreasing its concentration and effectiveness. EPI or blockers have grown as a potential and attractive strategy to overcome bacterial resistance on account of the dwindling novel antibiotics. Efflux pump blocker, Phenylalanyl arginyl β-naphthylamide (PAβN) has been widely explored for its potentiation activity in clinical pathogens possessing polyspecific efflux pumps (Lamers et al., 2013). 1-(1-napthylmethyl)-piperazine (NMP), another putative EPI was identified to potentiate oxacillin antibiotic by inhibiting direct binding of the substrate to the efflux pump (AcrAB and AcrEF) of pathogens in the *Enterobacteriaceae* family. However, the compound did not have action on *E. coli* isolates and MexAB-OprM or MexCD-OprJ efflux pumps of *P. aeruginosa* (Bohnert and Kern, 2005). Further, artesunate, an anti-malarial drug was identified to possess EPI activity by binding to AcrAB-TolC of *E. coli* and thereby potentiating several β-lactam antibiotics such as penicillin G, ampicillin, cefazolin, cefuroxime and cefoperazone (Li et al., 2011). A plant based 5,6,7-trihydroflavone EPI, Baicalein, was identified to potentiate β-lactam antibiotics including oxacillin, cefmetazole and ampicillin while Conessine, another plant-based compound interfered with MexAB-OprM efflux pump in MDR *P. aeruginosa* potentiating cefotaxime antibiotic (Siriyong et al., 2017). Karumathil et al., had identified the potentiation efficacy of *trans*-cinnamaldehyde, isolated from cinnamon, eugenol isolated from clove, carvacrol isolated from oregano oil and thymol obtained from thyme against MDR *A. baumanii*. The natural compounds exerted their action by acting on the AdeABC efflux pump of *A.baumanii* making it sensitive to seven β-lactam antibiotics toward which it was previously resistant (Karumathil et al., 2018). Catechin gallate, a component from tea extract, was identified to potentiate oxacillin against MRSA isolates by reducing their MIC from 64–512 μg mL^−1^ to ≤0.5–1 μg mL^−1^ (Stapleton et al., 2004). However, no EPIs which specifically potentiate β-lactam antibiotics have been commercialized and clinically used. Today, dual potentiation therapy by combining EPI-antibiotic combinations with antimicrobial photodynamic inactivation (APDI) that utilizes a photosensitizer to activate reactive oxygen species within the bacteria in presence of visible light and kill the pathogen are gaining popularity to target MDR and XDR strains (Tegos et al., 2008; Hamblin, 2016).

### 5.3. Outer membrane permeabilizers that potentiate β-lactam therapy

Permeabilizers are compounds that debilitate the OM thereby allowing the antibiotic to enter the bacterial cell and exert its action. Unlike the BLIs, research on β-lactam potentiators that act by permeabilizing OM and cell membranes of bacterial pathogens are at its infancy. There have been sparse studies that have identified OM permeabilizers that work in conjunction with β-lactam antibiotics though it is a promising strategy. Several natural compounds have been screened for its OM permeabilizing ability against β-lactamase producing and carbapenamase producing bacterial pathogens. Recently, Farrag et al. demonstrated the permeabilizing ability of gallic acid against β-lactamase producing *P. aeruginosa* (Farrag et al., 2019). The irreversible OM destabilization ability of gallic acid was determined to be the result of chelation of divalent cations from the OM and lipopolysaccharide components of the pathogen. A combinatorial potentiating strategy by combining an OM permeabilizing agent, a BLI and a suitable β-lactam antibiotic has been hypothesized to be an effective way to overcome infections caused by β-lactam resistant pathogens (Farrag et al., 2015). Interestingly, many of the BLIs that have been identified and clinically used also have PBP binding and modifying activity (Moya et al., 2017). Zidebactam and WCK 5153 have been identified to specifically bind to PBP2 apart from inhibiting class A, C and some class D β-lactamases which together enhance the activity of β-lactam antibiotic (Moya et al., 2017). Anti-parasite compound pentamidine was identified to potentiate Gram-positive drugs against the Gram-negative pathogen *A. baumanii* in animal models through a mechanism of membrane destabilization (Ando et al., 2012). Polymyxin antibiotics and nanopeptide derivatives of polymyxin B were identified to portray significant OM permeabilizing activity thereby potentiating β-lactam antibiotics (Vaara, 2019). Apart from the BLIs that exert OM permeabilizing activity, nanopeptide named NAB74 developed from polymyxin B has entered the initial phases of clinical trials making the strategy encouraging and optimistic.

### 5.4. Novel β-lactam potentiators

Apart from the classical β-lactam potentiators, many natural, synthetic, and semisynthetic compounds have been identified to potentiate β-lactam antibiotics against β-lactamase producing bacteria. Few promising β-lactam potentiators discovered within the last 5 years include farnesol, bulgecins, thiosemicarbazones and octyl gallate. Synthetic structural variants of farnesol, a sesquiterpene alcohol was identified to potentiate ampicillin and oxacillin against MRSA strains both *in-vitro* and *in-vivo* (Kim et al., 2018). Farnesol have been identified to upregulate and downregulate genes involved in cell membrane biogenesis and efflux pump proteins of Gram-negative pathogens *A. baumannii* and *E. coli* (Kostoulias et al., 2016). However, the exact molecular targets and mechanism by which these compounds potentiate β-lactams against MRSA are not elucidated. Bulgecins which are iminosaccharide secondary metabolites of *Paraburkholderia acidophila have been reported to be a potential* β-lactam enhancer against β-lactam resistant *P. aeruginosa* strains. The putative mechanism of ceftazidime and meropenem potentiation by bulgecin is understood to be the inhibition of periplasmic enzymes, Slt, MltD and MltG lytic transglycosylases that are very important for bacterial cell wall synthesis (Tomoshige et al., 2018). Additionally, diaryl and dipyridyl-substituted thiosemicarbazones were identified to possess synergistic effects with carbapenem antibiotic, meropenem against broad spectrum of MBL possessing bacterial pathogens (Zhao et al., 2021). Dipyridyl-substituted thiosemicarbazones competitively and reversibly inhibited NDM-1. The inhibition of MBLs by thiosemicarbazones was identified to be by the Zn(II) binding ability of a sulfur group in the compound (Li et al., 2021). Octyl gallate, a FDA approved antioxidant was identified to potentiate β-lactam antibiotics, penicillin, ampicillin and cephalothin against MRSA by increasing the bacterial cell wall permeability (Tamang et al., 2022). The galloyl moiety of octyl gallate is identified to interact with multiple targets in the bacterial cell wall which inhibit effective cell wall synthesis. Though the in-depth mechanistic evaluation of these molecules is still under evaluation, the differential targets of these compounds other than the conventional β-lactam drug targets make them potential futuristic β-lactam potentiators.

### 5.5. Non-conventional β-lactam antibiotic potentiators

#### 5.5.1. Antimicrobial peptides

Antimicrobial peptides (AMPs), also known as host defence peptides are positively charged short peptides found in a wide range of living organisms including bacteria. Against the backdrop of rapidly developing antibiotic resistance, attempts to put AMPs into clinical use as potentiators is accelerating. Thanatin, a single – disulfie-bond containing β-hairpin antimicrobial peptide was found to damage the OM of NDM-1-producing bacteria by displacing divalent cations competitively and promoting lipopolysaccharide leakage (Moura et al., 2020). Additionally, thanatin was identified to reduces NDM-1 enzymatic activity by displacing zinc ions from the active site of the enzyme and thereby restore carbapenem antibiotics against carbapenem resistant NDM-1-coding bacteria (Ma et al., 2019). Temporin A isolated from the skin excretions of the frog *Rana temporaria* was identified to potentiate imipenem and amoxicillin-clavulanic acid antibiotics and potentiate these antibiotics to re-sensitize MDR *E. faecalis* (Giacometti et al., 2005), Previously, Mandal et al., had created two novel short AMP BLIs, dBLIP-1 and dBLIP-2 by peptide rational designing technique which could potentiated β-lactam antibiotics (amoxicillin, ampicillin and cefotaxime) against plasmid mediated CTX-M-14 coding Gram-negative pathogens (Mandal et al., 2014). In another study employing natural AMPs having different structure and mode of action, PG-1, β-defensins, LL-37 were identified to have good membrane permeabilizing potential (Zharkova et al., 2019). Additionally, sub-inhibitory concentrations of a bivalent AMP, ASU014, used in combination with sub-inhibitory concentrations of oxacillin was identified to be effective against MRSA in both *in-vitro* and *in-vivo* studies (Lainson et al., 2017). Though AMPs are largely screened for discovering novel BLIs and OM permeabilizers, not many have been successfully translated clinically due to their host cell toxicity. To minimize toxicity and poor pharmacokinetics, AMPs are structurally modified that at many a times reduce its potency (Wang et al., 2021).

**Figure 3:**
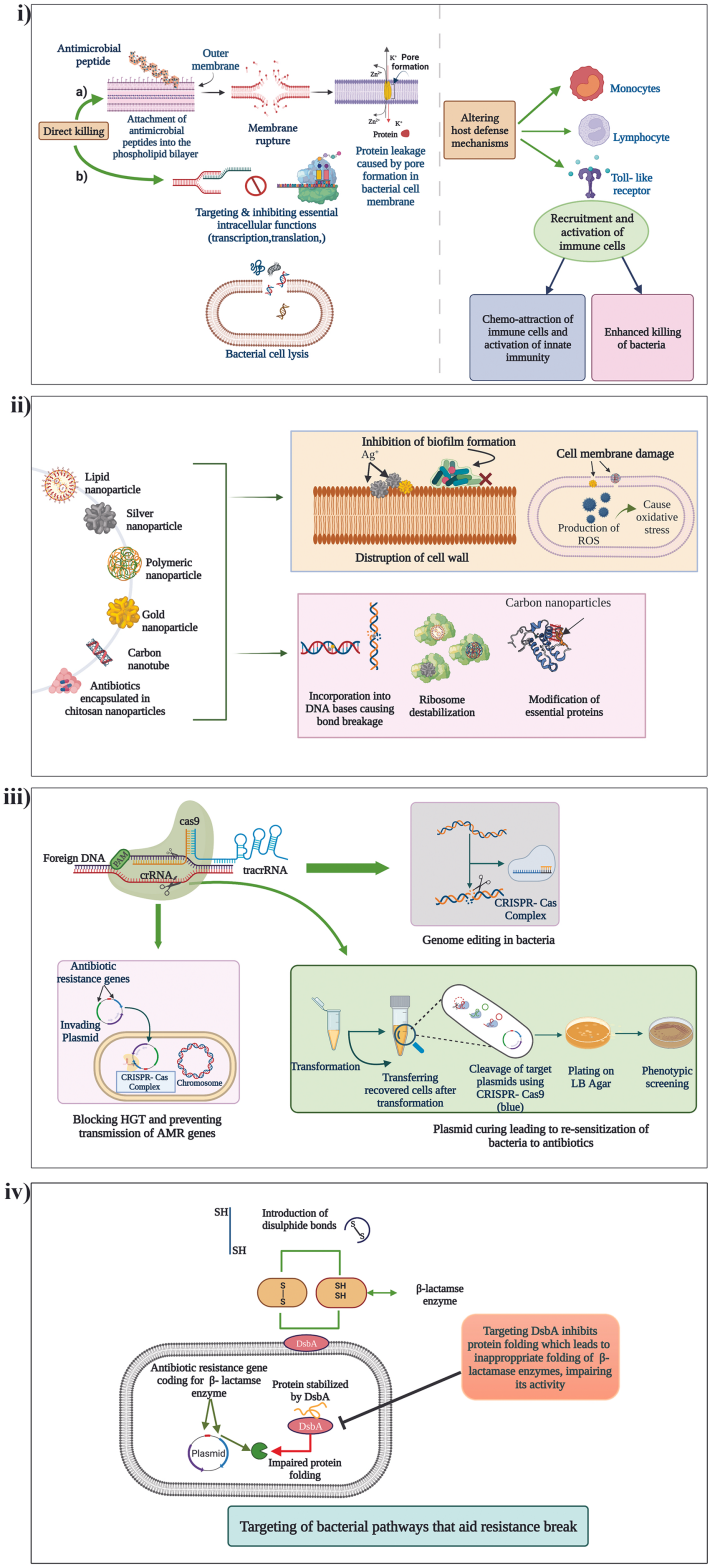
Mechanism of action of the different non-conventional antibiotic potentiators. (i) AMPs, (ii) Nanoparticles, (iii) CRISPR/Cas systems and (iv) Targeting of different bacterial pathways that aid in resistance break.

Peptidomimetics, which are synthetic analogues of peptides, capable of functionally being active and engaging in the same biological functions as that of AMPs, with reduced toxicity, is yet another popular method of antibiotic potentiation. Mood et al., described the potentiation activity of the peptidomimetic compound CEP-136 in combination with carbapenam antibiotics against carbapenamase producing clinical isolates *P. aeruginosa, K. pneumoniae*, *E. coli*, and *A. baumannii* (Mood et al., 2021). Yet in another study, a class of peptidomimetics were developed from human α-defensin 5 (HD5) which displayed promising potentiating activity of β-lactam antibiotics against MDR Gram-negative pathogens (Luo et al., 2022). The different mechanisms by which AMPs exert their potentiating activities is presented in Figure 3i.

#### 5.5.2. Nanoparticles

Nanoparticles (Nps) have long been investigated for their various therapeutic characteristics including antibacterial activity against drug resistant pathogens. Many Nps are known for their ability to penetrate the cell membranes of bacteria and interfere with their molecular functions thus devising unique antimicrobial activities (Mba and Nweze, 2021). Recent studies have also identified antibiotic potentiation ability of few Nps against β-lactam resistant pathogens. Graphene oxide and carbon nanotubes have displayed potent inhibitory effects on the MBL, VIM-2 by non-competitive adsorption of the enzyme to the surface of the Nps (Huang et al., 2015). In another study, silver nanoparticles (AgNPs) stabilized using bio-surfactin revealed good potentiation activity of amoxicillin, cefoperazone and doripenem against NDM-1, NDM-4, NDM-5, and NDM-7 producing *E. coli* (Kumar et al., 2019). AgNPs has also been identified to potentiate cefotaxime against ESBL producing *E. coli* (Mohammed et al., 2021). Furthermore, metal nanoparticles can be used as nanocarriers of antibiotics to minimize toxicity, and also lower the instances of resistance development through approaches of dual drug action which include antibiotic action and the nanoparticle activity targeting another pathway (Kotrange et al., 2021). Fan et al. encapsulated cephalosporin antibiotics (cefotaxime and ceftiofur) and BLIs (tazobactam and clavulanate) with cross-linked chitosan nanoparticles (CNAIs) which revealed excellent drug stability and antimicrobial activity against MDR ESBL-producing *Enterobacteriaceae* as compared to conventional antibiotic plus BLI therapy (Fan et al., 2022). Thus, nanoparticles carry a wide range of applications as potentiators and carriers that should be explored meticulously for therapeutic application. Mechanisms by which nanoparticles exert their potentiating activities is presented in Figure 3ii.

#### 5.5.3. CRISPR/Cas systems

Using CRISPR/Cas (Clustered regularly interspaced short palindromic repeats and CRISPR-associated protein) for countering AMR is one of the most exciting and latest strategies. The CRISPR/Cas system has the ability to degrade foreign incoming nucleic acids such as AMR gene encoding MGEs thereby acting as a bacterial immune system. CRISPR/Cas systems have shown potency to limit entry of plasmids into the bacterial cells and also cure them, which is explored to potentiate antibiotics (Butiuc-Keul et al., 2022). A study conducted by Tagliaferr et al., found a decreased expression of *bla*_TEM-1_ gene and decreased *bla*_TEM-1_ coding plasmid copy number in *E. coli* treated with CRISPR/Cas which thereby potentiated five β-lactam antibiotics (Tagliaferri et al., 2020). It was identified that the CRISPR/Cas system could cure even high copy number plasmids leading to complete sensitization of the antibiotics. In another study, Hao et al., recently developed a CRISPR/Cas system (pCasCure) that could re-sensitize carbapenem antibiotics against carbapenemase producing clinical isolates. The authors were able to obtain the deletion of plasmid mediated carbapenem resistance genes such as *bla*_KPC_*, bla*_NDM_ and *bla*_OXA-48_ by a rate of effectiveness of over 94 percent (Hao et al., 2020). Mechanisms by which CRISPR/Cas is utilized for potentiating antibiotics is presented in Figure 3iii. Though the strategy is effective *in-vitro*, the major challenge for its applicability in the clinical setting is the lack of an optimal deliver system of CRISPR-Cas9 into target bacterial populations.

#### 5.5.4. Identification of bacterial pathways that aid resistance break

New antibacterial agents, antibiotic potentiators and novel techniques to potentiate antibiotics are the need of the hour. Apart from the discovery of antibiotic adjuvants that either neutralize resistance enzymes or undermine cellular integrity, exploration of other bacterial pathways that aid in resistance break is becoming increasingly popular. DsbA is a protein present in bacteria which facilitates correct protein folding by presenting di-sulfide bonds leading to their stability. Furniss et al., investigated the role of DsbA modulation on incapacitating diverse β-lactamases in *E. coli* mutants. The study revealed that chemical inhibition of DsbA leads to resensitization of MDR Gram-negative pathogens to β-lactam antibiotics and increased the survival of animals challenged with MDR *P.aeruginosa* (Furniss et al., 2022). Similarly, experiments of Skredenske et al., had earlier demonstrated that targeting the global transcriptional activator, RhaS of XylS/AraC family proteins, substantially inhibited the transcription of various antibiotic resistance genes (Skredenske et al., 2013; Furniss et al., 2022). Screening of small molecule libraries against RhaS identified a highly selective compound able to potentiate various antibiotics. Thus, identification of novel unconventional potentiators of β-lactam antibiotics that target previously unrecognized targets have gained importance because of the evolution and diversification of β-lactam resistance genes. A representation of how targeting of a different bacterial pathway could aid in potentiating an antibiotic is presented in Figure 3iv.

## 6. β-Lactam potentiators-screening/identification strategies

There are various techniques by which a potential β-lactam potentiator could be screened and identified. In early times, for the identification of BLIs, screening was done by conventional agar well diffusion assays (Rolinson, 1991). However, recently, various less laborious techniques are developed for the fast, effective and accurate screening of large numbers of compounds. In the following section, we discuss the classical as well as emerging techniques used for the screening of BLIs and other potentiators that can reinstate the activity of β-lactam antibiotics.

### 6.1. Colorimetric assays for screening novel β-lactam potentiators

Colorimetric assays that analyze the growth inhibition of the pathogens in presence of antibiotic, compound and combinations of antibiotic and compounds is a conventional method of potentiator screening. Microwell plates are used to screen multiple compounds at a time. Reporter strains that over-express the enzyme or protein against which the compounds are being screened and chromogenic substrates that allow easy detection of the desired enzymatic activity are used to develop the assays. Compounds are initially screened for their potentiation activity and further assays are performed to characterize their mode of action. BLIs, EPIs and OM permeabilizers can be identified by this technique. Colorimetric assay with CENTA and Nitrocefin as chromogenic substrate revealed the antibacterial activity of *Syzygium aromaticum* oil and fresh juice of *Ocimum sanctum* leaves against ESBL producing *E. coli* (Shrivastav et al., 2019). A high throughput whole cell turbidity assay was used for screening a 645,000 compounds library against AmpC overproducing reporter strain to identify small molecule inhibitors of AmpG which is necessary for the induction of AmpC (Li et al., 2016). The study discovered 8 potential AmpG inhibitors that potentiate the activity of cefoxitin antibiotic inhibiting the growth of ESBL producing *P. aeruginosa* (Collia et al., 2018). Modified whole cell colorimetric assays such as the UV–Vis spectroscopy that can evaluate real time activity of β-lactamases and inhibitory activity of β-lactam potentiators are used widely for screening potential antibiotic adjuvants (Yang et al., 2017).

### 6.2. *In-silico* predictions

Molecular docking and molecular dynamic simulations (MDS) are very popular and well-established techniques for virtual screening of compounds. *In-silico* predictions not only predict the mechanism of resistance and identify novel potentiators, but also help in understanding structural restructuring capabilities for enhancing compound activity. During the screening of lead molecules, parameters of GRID Molecular Interaction Fields (MIFs) such as H-bond donor, H-bond acceptor and hydrophobicity are inspected to identify favorable active sites in the protein, and the molecule which gives the highest interactions is selected. Several studies have used *in-silico* screening to identify BLIs and EPIs that potentiate β-lactam antibiotics. FLAPdock (Fingerprint for Ligands and Proteins) is one such molecular docking tool that has identified a number of potential molecules active against class A β-lactamases (CTX-M, KPC-2, AmpC), class C β-lactamases, and MBLs (NDM-1 and VIM-2; Spyrakis et al., 2020). Ten natural compounds exhibiting potent MBL inhibiting activity targeting NDM-1 were identified by *in-silico* screening using Autodock Vina and Schrödinger suite software Extra Precision (XP; Spyrakis et al., 2020; Salari-Jazi et al., 2021). In another recent study, *in-silico* screening using Auto Dock4 software revealed Carsonic acid to exhibit promising inhibitory effects against NDM-1 (Yang et al., 2020). *In-silico* predictions thus are a promising strategy to identify broad-spectrum BLIs and EPIs though their *in-vitro* success has to be analyzed through various other assays.

### 6.3. Artificial intelligence – based screening

By being able to analyze massive amounts of data instantly, machine learning (ML), artificial intelligence (AI), and neural networks (NN) have ushered in a golden era of drug discovery and synthesis. Bhadra et al., had used ML to analyze distribution of amino acid sequences in AMPs to predict their novel antibacterial and potentiating activities (Bhadra et al., 2018; Cunha et al., 2021). Mansbach et al. used ML coupled with the Hunting Fox Algorithm to study the membrane penetration activity of compounds in MDR *P. aeruginosa* (Mansbach et al., 2020). ML integrated with high-throughput Fourier-transform infrared spectroscopy has been applied to detect novel β-lactam resistance genes and β-lactam potentiators (Cunha et al., 2021). Parvaiz et al. specifically screened compounds potentiating β-lactam antibiotics by targeting *bla*_CMY_ genes from a 700,000 compounds library by using Site-Identification by Ligand Competitive Saturation (SILCS) technology, Ligand-grid Free Energy (LGFE) analysis and ML based random-forest (RF) scoring (Parvaiz et al., 2021). Thus, the intersection of ML and AMR has enabled the development and enhancement of several models that have aided new antibiotic and antibiotic potentiator discovery.

### 6.4. Omics based approaches

In many cases, antibiotic potentiating molecules which are not synthetic are either derived from microorganisms itself such as secondary metabolites, plant based such as polyphenols or alkaloids or animal based such as AMPs. The presence of such antibiotic potentiating molecules could be detected by various omics techniques. The major omics techniques utilized for novel drug and potentiating molecule discovery include genomics, metagenomics, transcriptomics, proteomics and metabolomics.

- Genomics and metagenomics: Genomics techniques such as DNA microarray and next generation sequencing along with genome mining is useful in discovering biosynthetic gene clusters (BGCs) coding for novel bioactive molecules (Chandra Mohana et al., 2018). Many polyketides and non-ribosomal peptides with antibacterial and antibiotic potentiating activity has been identified through genome mining. As many as 17 novel BGCs coding for polyenes including salinilactam, a novel macrolactam molecule with potential antibacterial activity was identified through genome mining of marine actinomycete *Salinispora tropica* (Udwary et al., 2007). Likewise, several anti-infective molecules such as clarepoxcins, cypemycin, haloduracin, lactocillin, microcyclamide, teixobactin, reistomycin have been identified from different bacteria and actinomycetes through genomic approaches (Claesen and Bibb, 2010; Velásquez and van der Donk, 2011; Donia et al., 2014). The antibiotic potentiating activity of these molecules is worth exploring. Lactocillin was identified through metagenomic mining of human microbiome while clarepoxin was identified through multiplexed metagenomic mining of soil microbiome (Donia et al., 2014; Owen et al., 2015). Similarly, metagenomics coupled with synthetic biology approaches could be used to identify various non-ribosomal peptides with different bioactivities (Stevenson et al., 2019). Additionally, Santana-Pereira et al., had recently used shotgun sequencing of forest soil metagenomic libraries to identify 1015 BGCs (Santana-Pereira et al., 2020). Molecules from thus identified novel gene clusters could be screened for antibiotic potentiating activity. Hence, it is important to validate the expression of these gene clusters identified by genomics to understand the potency of biomolecules.
- Transcriptomics: Understanding the mode of action of prospective potentiating molecules is one of the major steps in developing the candidate molecule into a drug combination. Through transcriptomics, it is possible to identify and evaluate the molecule’s putative targets. Also, adverse drug target effects could be identified through transcriptomics (O'Rourke et al., 2020). Recently, Aunins and his team designed novel anti-sense inhibitors through transcriptomics-based approach which could potentiate carbapenem efficiency in carbapenem resistant *E. coli* (Aunins et al., 2020).
- Proteomics: Like the transcriptomics approach proteomics data is largely used to understand the dynamics of drug targets at the protein level upon candidate molecule treatment. A novel β-lactamase (Axc) with carbapenemase activity was identified through proteomics approach in a meropenem resistant clinical isolate of *Achromobacter xylosoxidans* (Fleurbaaij et al., 2018). Recently, Guan et al., had utilized proteomics to reveal synergistic action of theaflavin, a polyphenolic compound present in fermented tea with β-lactam antibiotics against MRSA (Guan et al., 2022). Cassiano et al., had recently utilized chemical proteomics guided approach to identify novel targets of the MBL inhibitor, magnolol (Cassiano et al., 2019). Proteomics could be thus used for understanding protein targets of synergistic and potentiating molecules. Most of the proteomics-based research studies in drug discovery have been performed to characterize protein expression profiling, functional proteomics and phosphoproteomics.
- Metabolomics: Identification of the metabolic pathways of the lead molecule through metabolomics has reduced the cost of toxicological screening. This has enabled improved clinical trials which has shortened the time needed for drugs to move through the development pipeline. Though metabolomics has been widely used to identify novel antibiotics, it is under explored for identifying β-lactam adjuvants (Tuyiringire et al., 2018; Aminov, 2022). Recently, Campos and Zampieri, had utilized high throughput metabolomic approach to test the activity of 1,279 pharmacologically diverse drugs with no known antibacterial activity on *E. coli*. The metabolome profiles of drug treated *E. coli* was compared with metabolome profiles of untreated *E. coli* to understand the activity of the small molecules. The study revealed nine drugs with non-promiscuous enzyme targets in the bacteria (Campos and Zampieri, 2019). Also Zampieri and his team had developed a rapid systemic metabolome profiling strategy to identify the different modes of action of new bioactive compounds (Zampieri et al., 2018).

## 7. Challenges of taking β-lactam potentiators from bench to bedside

**Figure 4:**
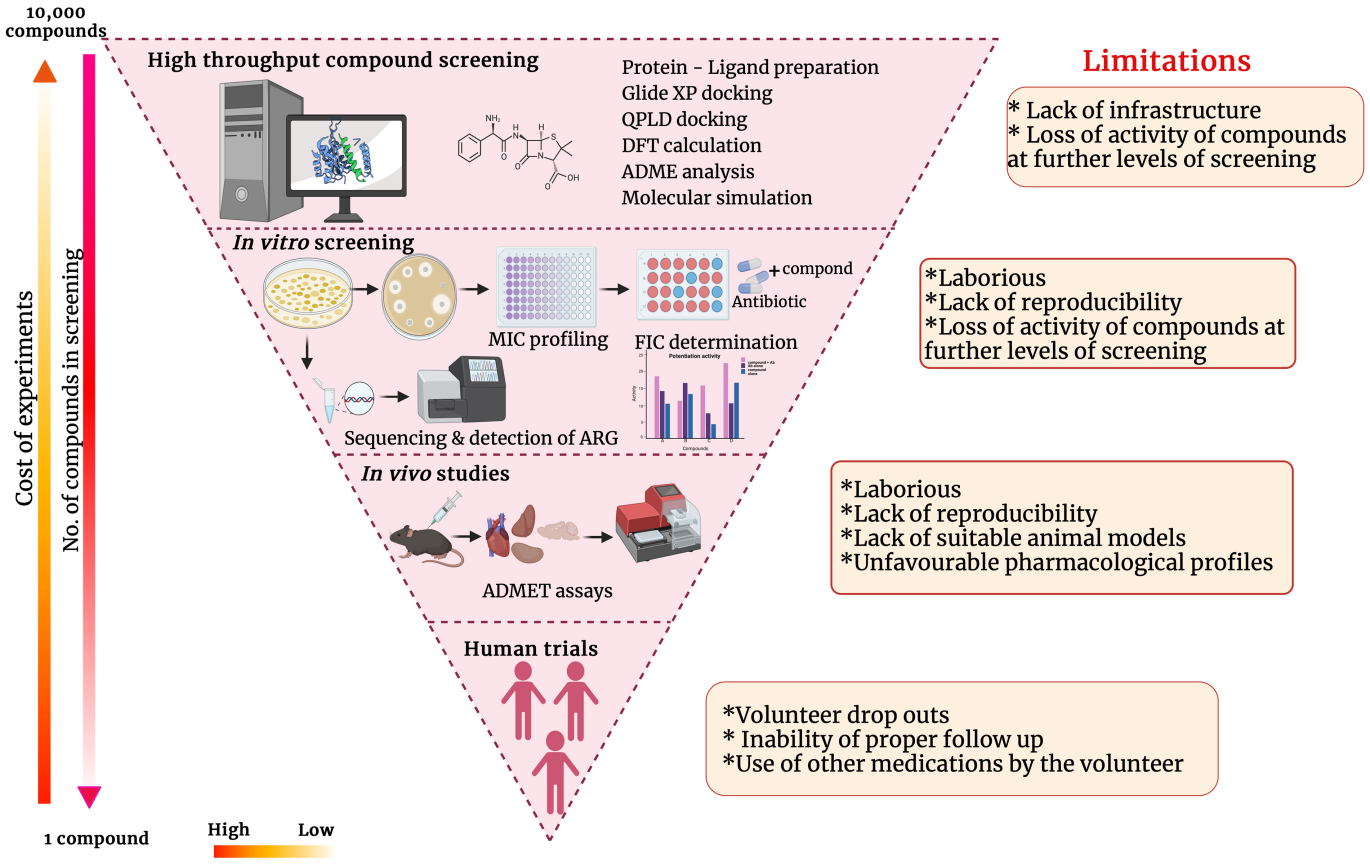
Inverted funnel plot representing the major processes of antibiotic potentiator discovery/development and the impediments at each stage.

Potentiators for sure obviate the urgent need to discover novel antibiotics. However, after the identification of a potential antibiotic adjuvant, development, and commercialization of it have a lot of roadblocks. Though the time taken and capital associated with novel antibiotic discovery and commercialization is considered higher than potentiator discovery, the process is invariable and equally strenuous. The major processes of antibiotic potentiator discovery/development and the impediments at each stage are represented in the inverted funnel plot (Figure 4). Though there have been many lead molecules identified as β-lactam potentiators through *in-silico* predictions, very few of them have been analyzed *in-vitro* and *in-vivo* on clinical pathogens to determine their efficacy. Automation of *in-vitro* screening and cutting down the labor would encourage more labs to translate the hit molecules they have identified through *in-silico* techniques to further levels. Interaction and collaboration between computational biologists and wet lab researchers can accelerate the hit molecule screening processes.

Various factors have to be considered before commercializing and using the antibiotic-antibiotic potentiator combination for therapeutic purposes. One major factor that has to be considered while combining the antibiotic and antibiotic potentiator is its compatibility. It has to be made sure that there are no unfavorable pharmacological profiles when the combination is used. For this, the concentrations of the antibiotic and antibiotic adjuvant have to be standardized. In many instances, it has been noticed that β-lactam-BLI combinations require a high concentration of either of the components for the desired activity in *in-vivo* studies, which can lead to toxicity or undesirable pharmacological outcomes (Papp-Wallace, 2019). Additionally, many BLIs exhibit low oral absorption, especially in their native form, and demand chemical modifications to stabilize them and exhibit better oral absorption (Luci et al., 2021). For instance, sulbactam in its native form was identified to have poor oral absorption as compared to pivaloyloxymethyl ester of sulbactam (Campoli-Richards and Brogden, 1987). Thus, structural modifications of the potentiator molecule could improve its absorption. Potentiator molecules both existing and newly identified could be used as the core to construct a structurally modified library which could be checked for improvement of activity at lower concentrations, improvement in absorption and other physiological parameters.

Other factors that have to be contemplated are target specificity and substrate specificity. Target specificity is very important, especially while developing EPI and it has to be ratified that it does not inhibit any of the eukaryotic efflux pumps. There are reports of many antibacterial and potentiating molecules that target both bacterial and human pathways (Dalhoff, 2021). Reserpine, known to be a potent Norfloxacin potentiator has been identified to have direct action as calcium antagonist on mammalian smooth muscle cells (Casteels and Login, 1983). Similarly, Verapamil, another mammalian calcium channel blocker has been identified to potentiate carbenicillin against *P. aeruginosa* (Hulen et al., 2020). Verampil is used to treat high blood pressure, angina and supraventricular tachycardia in humans. Further, it has been identified that many prokaryotic and eukaryotic enzymes share key structural features. Sevier et al., had previously revealed that the catalytic domain of eukaryotic oxidoreductases Ero1 and Erv2 share sequence homology and structural features similar to that of the catalytic core of DsbB (Sevier et al., 2005). Hence, molecules targeting DsbB catalytic core could also affect eukaryotic oxidoreductases which could hamper disulfide bond formation of eukaryotic peptides also. Thus, it is very important to develop molecules that have lower affinity to mammalian proteins and those which have higher specificity to prokaryotic targets. This could be possible by generating precise protein interaction networks and binding affinity studies of prokaryotic and eukaryotic targets with the lead molecule. Similarly, identification of a universal BLI that can target all β-lactamases is a herculean task and hence different combinations have to be used to treat different bacterial infections. Understanding the type of β-lactamases produced by the bacterial pathogens is warranted to determine the appropriate antibiotic-BLI combination. For the same, rigorous surveillance and documentation of different bacterial pathogens and their resistance profile both phenotypic and genotypic is necessary.

Another major problem of β-lactam potentiator commercialization is the rapid emergence of resistant microorganisms to them. Previously, more than 90% of isolates were susceptible to ampicillin-sulbactam combinations. But, now a days, extensive resistance of clinical pathogens against ampicillin-sulbactam has been reported (Ferreira et al., 2019). It has been only more than a decade that avibactam and relebactam potentiators were introduced for clinical use. However, bacterial pathogens especially the Gram-negatives have evolved mechanisms of resistance against them. Isolates harboring *bla*_KPC_ genes with specific mutations that confer DBO BLIs ineffective have been isolated from various parts of the world (Both et al., 2017; Giddins et al., 2018). There are various methods by which bacteria have developed methods to resist even β-lactam-BLI combinations. Overproduction of β-lactamase enzymes has been described as a major mechanism by which microorganisms resist β-lactam-BLI combinations. Additionally, mutations in the efflux pumps can reduce the potentiating action of EPIs. Schuster et al., had reported that random mutagenesis of AcrB efflux pump protein of *E. coli* resulted in reduced effectiveness of the most commonly used EPI, 1-(1-napthylmethyl)-piperazine (NMP; Schuster et al., 2014). To counteract this, molecules can be screened to target conserved protein domains in enzymes and efflux pumps that have lesser chance of resistance accumulations. Further, adaptive laboratory evolution studies of bacterial pathogens in the presence of the antibiotic and the lead molecule can be performed *in-vivo* and *in-vitro* to determine the possibilities of resistance developments.

## 8. Future directions and the way forward

Today, antibiotics are considered a double-edged sword. Due to the indiscriminate and inappropriate use of the antibiotics, bacterial pathogens have evolved multiple resistance mechanisms. To counteract the bacterial resistance and restore the efficacy of antibiotics, natural and synthetic compounds that do not possess antibacterial activity by themselves are being screened for their antibiotic potentiating activity. However, the vast structural and mechanical diversification of β-lactam resistance genes have caused bacterial pathogens to develop resistance to even the antibiotic-β-lactam potentiator combinations. In this context, screening of novel potential β-lactam potentiators to maintain the efficacy of this powerful class of antibiotics is highly warranted. Soon after the introduction of penicillin for clinical use in 1941, bacterial persistence was reported by Joseph Bigger in 1944 where he observed that a sub-population of *S. aureus* was not killed by penicillin. Today, persister population of bacteria cause a major cause of concern for clinicians as they cause treatment failures by alleviating bacterial resistance. Hence it is very important to identify an effective way to eradicate the persister population. A probable way would be identification of a β-lactam potentiators effective against persister bacterial population. Though there are no β-lactam potentiators found effective against persister bacterial populations, bithionol, an anthelmintic drug was identified to potentiate gentamicin against persister population of MRSA by increasing bacterial membrane permeability (Kim et al., 2019).

Natural and marine sources have been identified to serve as a vast resource for discovering novel bioactive compounds. Marine pharmacology is an expanding but underexplored field in which novel β-lactam potentiators could be discovered. The active interaction between the biotic and abiotic systems within the marine environment allows the marine flora and fauna to produce structurally and functionally distinct bioactive compounds. Multi-omics approaches like genomics, transcriptomics, proteomics and metabolomics can be integrated and used to discover potent β-lactam adjuvants. Machine learning and AI can be utilized to screen large datasets of known marine and natural bioactive molecules *in-silico*, apart from conventional *in-vitro* laboratory screening. Structural similarity to the existing β-lactam antibiotics can be one of the positive filtering options to identify a potent β-lactam potentiator. Furthermore, chemical or biological modifications of existing β-lactam potentiators to increase their efficacy when combined with antibiotics could be used to avoid the time-consuming process of screening and identifying potentiators from natural sources. Repurposing previously approved drugs for potentiation activity could also be used to develop effective β-lactam potentiators.

In addition to the screening of novel potentiators, ways to decrease the frantic use of antibiotics by means of non-antibiotic therapies have to be promoted to put a brake on the growing AMR burden. Awareness among the general public regarding the harmful effects of AMR is of prime importance to reduce antibiotic use. Traditional non-antibiotic therapies such as phage therapy and probiotic therapy could be utilized to treat infections. The specificity of phages to target specific bacteria could be utilized for effective pathogen elimination without disturbing the normal flora. Probiotic bacteria can be also used as a prophylactic agent as they either competitively exclude the pathogen from successful colonization, or create an unfavorable condition for the pathogen’s growth thereby preventing the infection. Furthermore, vaccines continue to be the most important non-antibiotic intervention that can prevent infections. Multiple vaccines have been developed against various pathogenic bacteria. Cocktails of β-lactamases that can create preventive immunity could be used to develop potential vaccine candidates to prevent infection by MDR pathogens.

## 9. Conclusion

Antibiotic therapy and resistance acquisition by microorganisms are two sides of the same coin. Since long, there have been limited potential antibiotics that have reached the market. The only option we are left with is to opt for novel antibiotic based and non-antibiotic based strategies that can improve the efficacy of existing antibiotics. Screening for antibiotic potentiators that can restore the activity of existing antibiotics is a promising strategy for the same. Though the screening of antibiotic potentiators are equally strenuous, it is considered to be a wiser and coherent option to limit bacterial resistance evolution to even last resort antibiotics.

## Author contributions

BD conceived the idea. LN collected the literature and drafted the review. MC contributed to the “β-lactam potentiators screening strategies” section and prepared Figure 3. SK and DP prepared the tables. All authors contributed to the article and approved the submitted version.

## Conflict of interest

The authors declare that the research was conducted in the absence of any commercial or financial relationships that could be construed as a potential conflict of interest.

## Publisher’s note

All claims expressed in this article are solely those of the authors and do not necessarily represent those of their affiliated organizations, or those of the publisher, the editors and the reviewers. Any product that may be evaluated in this article, or claim that may be made by its manufacturer, is not guaranteed or endorsed by the publisher.

## Figures and Tables

**Table:** 

ACA	Aminocephalosporinic acid
AI	Artificial intelligence
AMP	Antimicrobial peptides
AMR	Antimicrobial resistance
APA	Aminopenicillanic acid
APDI	Antimicrobial photodynamic inactivation
ATCC	American type culture collection
BGC	Biosynthetic Gene Clusters
BLDB	Beta-Lactamase DataBase
BLI	β-Lactamase inhibitors
CF	cystic fibrosis
CNAI	Cross-linked chitosan nanoparticles
CPE	Carbapenem-producing *Enterobacteriaceae*
CRISPR/Cas	Clustered regularly interspaced short palindromic repeats and CRISPR-associated protein
DBO	Diazabicyclooctanones
DHP-I	Dehydropeptidase-I
DTR	Difficult to treat
EPI	Efflux pump inhibitor
ESBL	Extended spectrum β-lactamase
FLAP	Fingerprint for Ligands and Proteins
ICU	Intensive care units
IS	Insertion sequences
LGFE	Ligand-grid Free Energy
MBL	Metallo-β-lactamases
MDR	Multidrug resistance
MDS	Molecular dynamic simulations
MGE	Mobile genetic elements
MIC	Minimum inhibitory concentration
MIF	Molecular Interaction Fields
ML	Machine learning
MRSA	Methicillin resistant *Staphylococcus aureus*
NMP	1-(1-napthylmethyl)-piperazine
NN	Neural networks
Nps	Nanoparticles
OM	Outer membrane
ORF	Open reading frame
PAβN	Phenylalanyl arginyl β-naphthylamide
PBP	Penicillin binding proteins
RF	Random-forest scoring
RND	Resistance-Nodulation-Division
SBL	serine β-lactamases
SILCS	Site-Identification by Ligand Competitive Saturation
UTI	Urinary tract infections
VAP	Ventilator associated pneumonia
WHO	World Health Organization
XP	Extra Precision
