# Supplementary figures and images for: β-Lactam potentiators to re-sensitize resistant pathogens: Discovery, development, clinical use and the way forward
Source: Front Microbiol. 2023 Mar 10;13:1092556. doi: 10.3389/fmicb.2022.1092556 (PMC10036598; doi:10.3389/fmicb.2022.1092556)

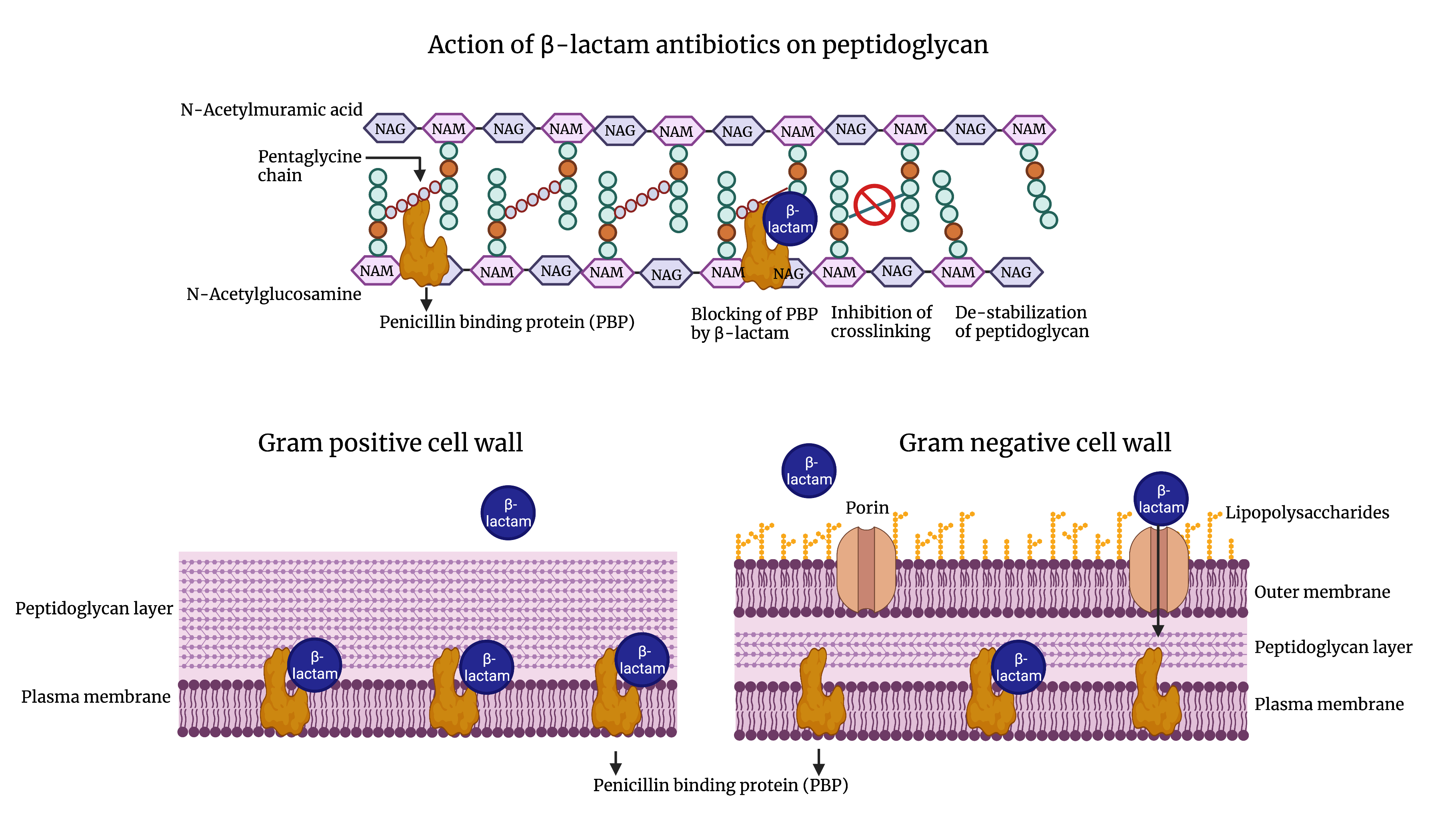

Supplement: Supplementary file 1 [file Image_1.JPEG]

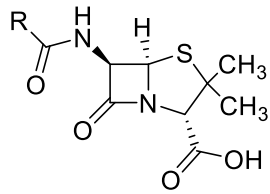

i) Penicillin

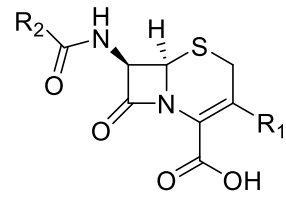

ii) Cephalosporin

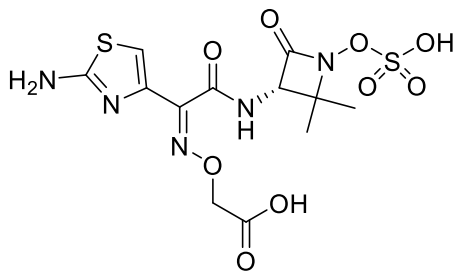

iii) Monobactam

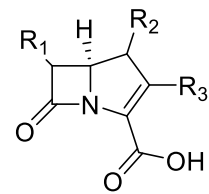

iv) Carbapenem

Supplement: Supplementary file 2 [file Image_2.PDF]
