# Supplementary material for: β-Lactam potentiators to re-sensitize resistant pathogens: Discovery, development, clinical use and the way forward
Source: Front Microbiol. 2023 Mar 10;13:1092556. doi: 10.3389/fmicb.2022.1092556 (PMC10036598; doi:10.3389/fmicb.2022.1092556)
Supplement: Supplementary file 3 [file Table_1.DOCX]

| **Penicillin** | | | | | | | |
| --- | --- | --- | --- | --- | --- | --- | --- |
| **Antibiotics** | **Source** | **Pharmaco-kinetics**  **(route of administration, absorption, route of elimination)** | **Spectrum** | **Resistance mechanism** | **Bacteria** | **Resistance determinants** | **References** |
| Penicillin G | Penicillium  chrysogenum | Intravenous,  Intramuscular  Rapidly absorbed  High serum concentration  Elimination via urine and biliary excretion | Narrow spectrum | Modification in PBPs  Or Expressions of penicillin-binding protein | *Enterococcus faecium* | Class A PBPs PbpF, PonA or PbpZ genes.  pbp5 gene encodes class -B- PBP;  pbp4 gene (V223I, A617T) | Rice et al., 2001, 2018; Signoretto et al., 1994 |
| Penicillin V | Penicillium notatum | Oral  Rapidly but incompletely absorbed  Elimination via urine | Broad spectrum | Hyperproduction of β-lactamases | *Enterococcus faecium* | blaZ gene | Miller et al., 2014; Kernodle et al., 2006; Lowy et al., 2003 |
| Ampicillin  (Penicillinase resistant penicillin) | Semisynthetic | Oral, Intravenous  Well absorbed from gastrointestinal (GI) tract  Eliminated via feces and bile | Broad spectrum | Mutation or overproduction of PBP-5, Expressions of penicillin-binding protein, mutation in *ftsI* gene | *Enterococcus faecium, Klebsiella* sp*., Enterobacter* sp.*, Acinetobacter* sp. | TEM-1, ROB-1 | Zhang et al., 2012;  [Greer et al., 2008](https://paperpile.com/c/whZSUZ/yGL7+ngbC) |
| Oxacillin  (Penicillinase resistant penicillin) | Semisynthetic | Oral, Intravenous  Low absorption rate  Rapidly eliminated via urine | Narrow spectrum | Hyperproduction of β-lactamases, Alteration of PBPs | *Staphylococcus aureus, Enterococcus* sp*., Klebsiella* sp*., Enterobacter* sp.*, Acinetobacter* sp. | OXA-β lactamases (ESBL & Carbapenemase); mecA | Livermore et al., 1995;  Philippon et al., 2002;  Philippon et al., 2002;  Kubota et al., 2018;  Bratu et al., 2005;  Ho et al., 2002;  Szabó et al., 2005 |
| Amoxicillin  (Aminopenicillin) | Semisynthetic | Oral  60% bioavailability  Eliminated via urine after 6 hours | Broad spectrum |  | *Escherichia coli, Klebsiella pneumoniae, Enterobacter* sp*.* | Production of β -lactamases |  |
| Dicloxacillin  (Penicillinase resistant penicillin) | Semisynthetic | Oral  Rapidly but incompletely absorbed  Route of elimination not studied in detail | Narrow spectrum |  | *Staphylococcus aureus* | Production of β -lactamases |  |
| Cloxacillin  (Penicillinase resistant penicillin) | Semisynthetic | Oral  Well absorbed from GI tract  Route of elimination not studied in detail | Narrow spectrum |  | *Staphylococcus aureus* |  |  |
| Methicillin | Semisynthetic | Intravenous, Intramuscular  Moderate absorption  Route of elimination not studied in detail | Narrow spectrum |  | *Staphylococcus aureus* | mecA, mecC | Arede et al., 2013; Paterson et al., 2014 |
| Nafcillin  (Penicillinase resistant penicillin) | Semisynthetic | Intravenous  Moderate absorption  Elimination via non-renal route | Narrow spectrum |  | *Staphylococcus aureus* | Mutation in *vraRST* operon | Bagcigil et al., 2012;  Malachowa and DeLeo, 2010  Hamoudi et al., 1983; Salazar  et al., 2020;  Shaokat et al., 1995;  [Verbist and Verhaegen 1986](https://paperpile.com/c/whZSUZ/PVEN);  Hubbard et al., 2020 |
| Azlocillin | Semisynthetic | Oral  Not significantly absorbed from the GI tract  Route of elimination not studied in detail | Narrow spectrum |  | *Klebsiella* sp. | TEM |  |
| Carbenicillin  (Extended spectrum penicillin) | Semisynthetic | Oral  Rapidly absorbed from the small intestine  Route of elimination not studied in detail | Extended spectrum |  | *Enterobacter cloacae, Klebsiella pneumoniae* | TEM-1, TEM-2, SHV-1 |  |
| Ticarcillin  (Extended spectrum penicillin) | Semisynthetic | Intravenous  Absorbance and clearance not significantly studied | Extended spectrum |  | *Enterococcus* sp*., Klebsiella* sp*., Acinetobacter* sp.,  *Enterobacter* sp., | Production of inducible β-lactamases |  |
| Piperacillin  (Extended spectrum penicillin) | Semisynthetic | Intravenous  Low absorption rate  Largely not metabolized and rapidly eliminated via urine and biliary route | Extended spectrum |  | *Acinetobacter* spp*., Klebsiella* sp*.* | TEM-1B |  |
| Mezlocillin  (Extended spectrum penicillin) | Semisynthetic | Intravenous  Low absorption rate  Eliminated via urine or biliary route | Extended spectrum |  | *Penicillinase* producing organism*, Klebsiella pneumoniae, Enterobacter* sp. | TEM-1 |  |

| **Cephalosporin C**  **(Natural- Acremonium chrysogenum)** | | | | | | | |
| --- | --- | --- | --- | --- | --- | --- | --- |
| **Antibiotics** | **Source** | **Pharmaco-kinetics**  **(route of administration, absorption, route of elimination)** | **Spectrum** | **Resistance mechanism** | **Bacteria** | **Resistance determinants** | **References** |
| **1^st^ generation**  Cefadroxcil  Cephalexin  Cephradin  Cephalothin  Cephapirin  Cefazolin  Cefecetrile  Cefaloglycin  Cefroxadine  Cefaloridine  Cefradine  **2^nd^ generation**  Cefamandole  Cefanicid  Cefmetazole Cefachlor  Cefotetan  Cefuroxime  **3^rd^ generation**  Cefixime  Cefotaxime Cefodoxime  Ceftibutin  Cefoperazone  Ceftizoxime  Ceftriaxone  Ceftazidime  Moxalacatm  Cefoxitin  **4^th^ generation**  Cefepime  Cefozopran  Cefpirome  Cefquinome Ceftobiprole  **5^th^ generation**  Cefdinir  Ceftolozane  Ceftaroline  Ceftobiprole  Fosamil  (Ceftaroline fosamil) | **Semisynthetic**  **Semisynthetic**  **Semisynthetic**  **Semisynthetic**  **Semisynthetic** | The degree of Cephalosporin C metabolism and absorption varies  Few cephalosporins have a high biliary elimination  **Oral** Cephalexin  Cefdinir  Cefachlor  Cefixime  Cefodoxime  Ceftibutin  Cefuroxime  **Intravenous**  Cefazolin  Cefuroxime  Cefotaxime  Cefozopran  Ceftriaxone  Ceftazidime  Cefepime  (Ceftaroline fosamil)  Moxalactam  Cefoxitin  Ceftobiprole  Ceftolozane | Most Gram-positive, some Gram-negative bacteria  **Narrow spectrum**  Cephalexin  Cephradin  **Broad spectrum**  Cefadroxcil  Cefuroxime  cefdinir,  cefixime  Cephalothin  Cephapirin  Cefazolin  Cefecetrile  Cefepime  Cefaloglycin  (no longer in use) | Mutation in penicillin-binding proteins (PBPs) | *Enterococcus faecium* | PBP5  PBP4 (T418A),  (L475Q)  Class A PBPs.  PBPF and PonA | Arbeloa et al., 2004;  Kristich et al., 2011  Lazzaro et al., 2022 |
|  |  |  |  | Hyperproduction of β-lactamases  Bacterial two-component regulatory systems (TCS) | *Enterococcus faecium* | CTX-M-2  CroRS (serine/threonine kinase designated IreK a phosphatase IreP  and IreB) | Comenge  et al.,  2003;  Hall et al., 2013 |
|  |  |  |  | Hyperproduction of β-lactamases | Enterobacter   sp. | Extended spectrum  AmpC  (ESAC)  β**-**lactamases | Pai et al., 2004 |
|  |  |  |  | Hyperproduction of β-lactamases | *Klebsiella pneumoniae* | Extended spectrum  Class A TEM, SHV, CTX type β-lactamases (CMY -19, CTX-M-14, CTX-M-15, CTX-M-33, KPC-2, Class D ESBL oxacillinases | Wachino et al., 2006;  Liakopoulos et al., 2016;  Ogbolu et al., 2018 |
|  |  |  |  | Hyperproduction of β-lactamases | *Pseeudomonas aeruginosa* | Class A(CTX-M-15, SHV), Class C- AmpC, Class D (OXA-10, OXA-11,  OXA-15) and VEB -1 | Hall et al., 1993;  Danel et al 1997;  Ogbolu et al., 2018 |
|  |  |  |  | Hyperproduction of β-lactamases | *Acinetobacter baumannii* | Class C (ADC) | Hujer et al., 2005;  Joshi et al., 2017 |

| **Carbapenems** | | | | | | | | |
| --- | --- | --- | --- | --- | --- | --- | --- | --- |
| **Antibiotics** | **Source** | **Pharmaco-kinetics**  **(route of administration, absorption, route of elimination)** | **Spectrum** | **Resistance mechanism** | **Bacteria** | **Resistance determinants** | **References** |  |
| Imipenem | Semisynthetic | Intravenous administration  Not effectively absorbed by the GI tract  Eliminated via urine | Broad spectrum  activity against both Gram-positive and Gram-negative bacteria | Mutation in PBPs,  Hyperproduction of PBPs | *Enterococcus faecium* | PBP4  (P520S and Y605H);  Overproduction of PBP5 | Amin et al., 2001; Ono et., al  2005 |  |
|  |  |  |  | Hyperproduction of β-lactamases | *Klebsiella pneumoniae* | Class A β-lactamases (KPC, CTX-M)  MBLs (NDM-1, IMP-1, VIM-1,  BKC-1) | Nordmannet al., 2009;  Bonnin et al., 2021 |  |
|  |  |  |  | Hyperproduction of β-lactamases | *Acinetobacter baumannii* | OXA-23, OXA-40, OXA-58,  ADC-7 | Alsultan, et al., 2009; Hujer  et al., 2005 |  |
|  |  |  |  | Hyperproduction of β-lactamases  Overexpression of efflux pump | *Pseudomonas aeruginosa* | Class A (GES-5), MBL (VIM-1, VIM-2, IMP-1, IMP-6, IMP-9, AIM-1  MexAB-OprM, OprD | Amsalu et al., 2021; Ma et al., 2021 |  |
|  |  |  |  | Hyperproduction of β-lactamases | *Enterobacter cloacae* | MBL (LMB-1, Linz Metallo-β-lactamase) | Lange et al., 2018 |  |
| Ertapenem  Meropenem  Doripenem | Semisynthetic | Intravenous  The degree of metabolism and absorption varies  Elimination is via urine | Broad spectrum activity against aerobic Gram-negative bacteria | Hyperproduction of β-lactamases  Lower expression of efflux pump | *Klebsiella pneumoniae* | CTX-M-15  OmpK35, OmpK36 porin variant | Poulou et al., 2013 |  |
| Biapenem  Panipenem  (Only licensed in Japan) | Semisynthetic | Intravenous  The degree of metabolism and absorption varies  Elimination is via urine | Broad spectrum  Gram-negative and Gram-positive aerobic and anaerobic bacteria | Not reported | Unknown | Unknown | **-** |  |
| Tebipenem  /pivoxil  (Under clinical trials) | Semisynthetic | Oral  Rapid absorption on oral administration  Elimination is via urine | Broad spectrum  Gram-negative and Gram-positive aerobic and anaerobic bacteria Broad spectrum | Not reported | Unknown | Unknown | **-** |  |

| **Monobactam** | | | | | | | |
| --- | --- | --- | --- | --- | --- | --- | --- |
| **Antibiotics** | **Source** | **Pharmaco-kinetics**  **(route of administration, absorption, route of elimination)** | **Spectrum** | **Resistance mechanism** | **Bacteria** | **Resistance determinants** | **References** |
| Aztreonam (only clinically available monobactam) | Semi-synthetic | Intravenous/ intramuscular/nebulized/ intraperitoneal;  Poor absorption on oral administration, rapidly absorbed on intramuscular administration  Eliminated via urine | Aerobic Gram-negative organisms | Hyperproduction of β-lactamases | Klebsiella pneumoniae | TEM-7, CMY-16 (Y150S, N346H) | Arlet et al, 1993 |
|  |  |  |  | Hyperproduction of β-lactamases | *Enterobacter cloacae* | AmpD, AmpC, | Annavajhala et al, 2019 |
|  |  |  |  | Mutations in the efflux pump transcription regulators, Overexpression of efflux pump | *Pseudomonas*  *aeruginosa* | MexR (R70Q), NalC(R97G, A186T), NalD, MexAb-OprM | Braz et al, 2016, Jorth et al, 2017, Yan et al, 2019, Ma et al, 2021 |
| Tigemonam | Synthetic | Oral  Rapid absorption on oral administration with 84% bioavailability  Eliminated via urine | Aerobic Gram-negative organisms, not effective against Pseudomonas and Acinetobacter sp. | Hyperproduction of β-lactamases | Aztreonam resistant Enterobacter | Unknown | Chin et al, 1988 |
| Carumonam | Synthetic | Intravenous  Not rapidly absorbed on oral administration  Excreted unchanged in urine | Aerobic Gram-negative organisms including Pseudomonas sp, | Not reported | Unknown | Unknown | - |
| Nocardicin A | Nocardia uniformis | Intravenous / intramuscularly  Rapid absorption of 65-80% on intravenous injection  Elimination via urine | Broad-spectrum of Gram-negative bacteria including Proteus and Pseudomonas | Not reported | Unknown | Unknown | - |

**Supplementary Table 1.** List of major classes of β-lactam antibiotics, their spectrum of activity, major molecular targets and resistance mechanisms in *ESKAPE* pathogens.

[Alsultan, A. A., Hamouda, A., Evans, B. A., and Amyes, S. G. B. (2009). *Acinetobacter baumannii*: Emergence of Four Strains with Novel*bla*_OXA-51-like_Genes in Patients with Diabetes Mellitus. *Journal of Chemotherapy* 21, 290–295. doi:](http://paperpile.com/b/whZSUZ/wbuQ) [10.1179/joc.2009.21.3.290](http://dx.doi.org/10.1179/joc.2009.21.3.290)[.](http://paperpile.com/b/whZSUZ/wbuQ)

[Amin, N. E. L., El Amin, N., Lund, B., Tjernlund, A., Lundberg, C., Jalakas, K., et al. (2001). Mechanisms of resistance to imipenem in imipenem-resistant, ampicillin-sensitive Enterococcus faecium. *APMIS* 109, 791–797. doi:](http://paperpile.com/b/whZSUZ/ElNg) [10.1034/j.1600-0463.2001.d01-148.x](http://dx.doi.org/10.1034/j.1600-0463.2001.d01-148.x)[.](http://paperpile.com/b/whZSUZ/ElNg)

[Annavajhala, M. K., Gomez-Simmonds, A., and Uhlemann, A.-C. (2019). Multidrug-Resistant Enterobacter cloacae Complex Emerging as a Global, Diversifying Threat. *Frontiers in Microbiology* 10. doi:](http://paperpile.com/b/whZSUZ/Dt9o) [10.3389/fmicb.2019.00044](http://dx.doi.org/10.3389/fmicb.2019.00044)[.](http://paperpile.com/b/whZSUZ/Dt9o)

[Arbeloa, A., Segal, H., Hugonnet, J.-E., Josseaume, N., Dubost, L., Brouard, J.-P., et al. (2004). Role of Class A Penicillin-Binding Proteins in PBP5-Mediated β-Lactam Resistance in *Enterococcus faecalis*. *Journal of Bacteriology* 186, 1221–1228. doi:](http://paperpile.com/b/whZSUZ/IoAL) [10.1128/jb.186.5.1221-1228.2004](http://dx.doi.org/10.1128/jb.186.5.1221-1228.2004)[.](http://paperpile.com/b/whZSUZ/IoAL)

[Arêde, P., Ministro, J., and Oliveira, D. C. (2013). Redefining the Role of the β-Lactamase Locus in Methicillin-Resistant Staphylococcus aureus: β-Lactamase Regulators Disrupt the MecI-Mediated Strong Repression on *mecA* and Optimize the Phenotypic Expression of Resistance in Strains with Constitutive *mecA* Expression. *Antimicrobial Agents and Chemotherapy* 57, 3037–3045. doi:](http://paperpile.com/b/whZSUZ/y4K5) [10.1128/aac.02621-12](http://dx.doi.org/10.1128/aac.02621-12)[.](http://paperpile.com/b/whZSUZ/y4K5)

[Arlet, G., Rouveau, M., Fournier, G., Lagrange, P. H., and Philippon, A. (1993). Novel, plasmid-encoded, TEM-derived extended-spectrum beta-lactamase in Klebsiella pneumoniae conferring higher resistance to aztreonam than to extended-spectrum cephalosporins. *Antimicrobial Agents and Chemotherapy* 37, 2020–2023. doi:](http://paperpile.com/b/whZSUZ/nVGr) [10.1128/aac.37.9.2020](http://dx.doi.org/10.1128/aac.37.9.2020)[.](http://paperpile.com/b/whZSUZ/nVGr)

[Bagcigil, A. F., Taponen, S., Koort, J., Bengtsson, B., Myllyniemi, A.-L., and Pyörälä, S. (2012). Genetic basis of penicillin resistance of S. aureus isolated in bovine mastitis. *Acta Veterinaria Scandinavica* 54. doi:](http://paperpile.com/b/whZSUZ/OCAO) [10.1186/1751-0147-54-69](http://dx.doi.org/10.1186/1751-0147-54-69)[.](http://paperpile.com/b/whZSUZ/OCAO)

[Bonnin, R. A., Jousset, A. B., Emeraud, C., Oueslati, S., Dortet, L., and Naas, T. (2020). Genetic Diversity, Biochemical Properties, and Detection Methods of Minor Carbapenemases in Enterobacterales. *Front. Med.* 7, 616490.](http://paperpile.com/b/whZSUZ/qEmu)

[Bratu, S., Landman, D., Alam, M., Tolentino, E., and Quale, J. (2005). Detection of KPC Carbapenem-Hydrolyzing Enzymes in *Enterobacter* spp. from Brooklyn, New York. *Antimicrobial Agents and Chemotherapy* 49, 776–778. doi:](http://paperpile.com/b/whZSUZ/YdV8) [10.1128/aac.49.2.776-778.2005](http://dx.doi.org/10.1128/aac.49.2.776-778.2005)[.](http://paperpile.com/b/whZSUZ/YdV8)

[Braz, V. S., Furlan, J. P. R., Fernandes, A. F. T., and Stehling, E. G. (2016). Mutations in NalC induce MexAB-OprM overexpression resulting in high level of aztreonam resistance in environmental isolates of*Pseudomonas aeruginosa*. *FEMS Microbiology Letters* 363, fnw166. doi:](http://paperpile.com/b/whZSUZ/mbwZ) [10.1093/femsle/fnw166](http://dx.doi.org/10.1093/femsle/fnw166)[.](http://paperpile.com/b/whZSUZ/mbwZ)

[Chin, N. X., and Neu, H. C. (1988). Tigemonam, an oral monobactam. *Antimicrobial Agents and Chemotherapy* 32, 84–91. doi:](http://paperpile.com/b/whZSUZ/5nbT) [10.1128/aac.32.1.84](http://dx.doi.org/10.1128/aac.32.1.84)[.](http://paperpile.com/b/whZSUZ/5nbT)

[Comenge, Y., Quintiliani, R., Li, L., Dubost, L., Brouard, J.-P., Hugonnet, J.-E., et al. (2003). The CroRS Two-Component Regulatory System Is Requiredfor Intrinsic β-Lactam Resistance in *Enterococcusfaecalis*. *Journal of Bacteriology* 185, 7184–7192. doi:](http://paperpile.com/b/whZSUZ/V4Mc) [10.1128/jb.185.24.7184-7192.2003](http://dx.doi.org/10.1128/jb.185.24.7184-7192.2003)[.](http://paperpile.com/b/whZSUZ/V4Mc)

[Danel, F., Hall, L. M., Gur, D., and Livermore, D. M. (1997). OXA-15, an extended-spectrum variant of OXA-2 beta-lactamase, isolated from a Pseudomonas aeruginosa strain. *Antimicrobial Agents and Chemotherapy* 41, 785–790. doi:](http://paperpile.com/b/whZSUZ/IeUD) [10.1128/aac.41.4.785](http://dx.doi.org/10.1128/aac.41.4.785)[.](http://paperpile.com/b/whZSUZ/IeUD)

[Greer, L. G., Roberts, S. W., Sheffield, J. S., Rogers, V. L., Hill, J. B., Mcintire, D. D., et al. (2008). Ampicillin resistance and outcome differences in acute antepartum pyelonephritis. *Infect. Dis. Obstet. Gynecol.* 2008, 891426.](http://paperpile.com/b/whZSUZ/ngbC)

[Hall, C. L., Tschannen, M., Worthey, E. A., and Kristich, C. J. (2013). IreB, a Ser/Thr Kinase Substrate, Influences Antimicrobial Resistance in Enterococcus faecalis. *Antimicrobial Agents and Chemotherapy* 57, 6179–6186. doi:](http://paperpile.com/b/whZSUZ/lt16) [10.1128/aac.01472-13](http://dx.doi.org/10.1128/aac.01472-13)[.](http://paperpile.com/b/whZSUZ/lt16)

[Hall, L. M., Livermore, D. M., Gur, D., Akova, M., and Akalin, H. E. (1993). OXA-11, an extended-spectrum variant of OXA-10 (PSE-2) beta-lactamase from Pseudomonas aeruginosa. *Antimicrobial Agents and Chemotherapy* 37, 1637–1644. doi:](http://paperpile.com/b/whZSUZ/kXbR) [10.1128/aac.37.8.1637](http://dx.doi.org/10.1128/aac.37.8.1637)[.](http://paperpile.com/b/whZSUZ/kXbR)

[Hamoudi, A. C., Palmer, R. N., and King, T. L. (1983). Nafcillin Resistant Staphylococcus Aureus A Possible Community Origin. *Infection Control* 4, 153–157. doi:](http://paperpile.com/b/whZSUZ/LrU5) [10.1017/s0195941700058070](http://dx.doi.org/10.1017/s0195941700058070)[.](http://paperpile.com/b/whZSUZ/LrU5)

[Ho, P. L., Shek, R. H. L., Chow, K. H., Duan, R. S., Mak, G. C., Lai, E. L., et al. (2005). Detection and characterization of extended-spectrum β-lactamases among bloodstream isolates of Enterobacter spp. in Hong Kong, 2000–2002. *Journal of Antimicrobial Chemotherapy* 55, 326–332. doi:](http://paperpile.com/b/whZSUZ/a51U) [10.1093/jac/dki010](http://dx.doi.org/10.1093/jac/dki010)[.](http://paperpile.com/b/whZSUZ/a51U)

[Hubbard, A. T. M., Mason, J., Roberts, P., Parry, C. M., Corless, C., van Aartsen, J., et al. (2020). Piperacillin/tazobactam resistance in a clinical isolate of Escherichia coli due to IS26-mediated amplification of blaTEM-1B. *Nature Communications* 11. doi:](http://paperpile.com/b/whZSUZ/Sd14) [10.1038/s41467-020-18668-2](http://dx.doi.org/10.1038/s41467-020-18668-2)[.](http://paperpile.com/b/whZSUZ/Sd14)

[Hujer, K. M., Hamza, N. S., Hujer, A. M., Perez, F., Helfand, M. S., Bethel, C. R., et al. (2005). Identification of a New Allelic Variant of the *Acinetobacter baumannii* Cephalosporinase, ADC-7 β-Lactamase: Defining a Unique Family of Class C Enzymes. *Antimicrobial Agents and Chemotherapy* 49, 2941–2948. doi:](http://paperpile.com/b/whZSUZ/iFo2) [10.1128/aac.49.7.2941-2948.2005](http://dx.doi.org/10.1128/aac.49.7.2941-2948.2005)[.](http://paperpile.com/b/whZSUZ/iFo2)

[Jorth, P., McLean, K., Ratjen, A., Secor, P. R., Bautista, G. E., Ravishankar, S., et al. (2017). Evolved Aztreonam Resistance Is Multifactorial and Can Produce Hypervirulence in. *MBio* 8. doi:](http://paperpile.com/b/whZSUZ/K2VA) [10.1128/mBio.00517-17](http://dx.doi.org/10.1128/mBio.00517-17)[.](http://paperpile.com/b/whZSUZ/K2VA)

[Joshi, P. R., Acharya, M., Kakshapati, T., Leungtongkam, U., Thummeepak, R., and Sitthisak, S. (2017). Co-existence of bla OXA-23 and bla NDM-1 genes of Acinetobacter baumannii isolated from Nepal: antimicrobial resistance and clinical significance. *Antimicrobial Resistance & Infection Control* 6. doi:](http://paperpile.com/b/whZSUZ/wu8B) [10.1186/s13756-017-0180-5](http://dx.doi.org/10.1186/s13756-017-0180-5)[.](http://paperpile.com/b/whZSUZ/wu8B)

[Kernodle, D. S. (2014). Mechanisms of Resistance to β-Lactam Antibiotics. *Gram-Positive Pathogens*, 769–781. doi:](http://paperpile.com/b/whZSUZ/qpbN) [10.1128/9781555816513.ch62](http://dx.doi.org/10.1128/9781555816513.ch62)[.](http://paperpile.com/b/whZSUZ/qpbN)

[Kristich, C. J., Little, J. L., Hall, C. L., and Hoff, J. S. (2011). Reciprocal Regulation of Cephalosporin Resistance in Enterococcus faecalis. *mBio* 2. doi:](http://paperpile.com/b/whZSUZ/Sln3) [10.1128/mbio.00199-11](http://dx.doi.org/10.1128/mbio.00199-11)[.](http://paperpile.com/b/whZSUZ/Sln3)

[Kubota, H., Uwamino, Y., Matsui, M., Sekizuka, T., Suzuki, Y., Okuno, R., et al. (2018). FRI-4 carbapenemase-producing Enterobacter cloacae complex isolated in Tokyo, Japan. *Journal of Antimicrobial Chemotherapy* 73, 2969–2972. doi:](http://paperpile.com/b/whZSUZ/8utR) [10.1093/jac/dky291](http://dx.doi.org/10.1093/jac/dky291)[.](http://paperpile.com/b/whZSUZ/8utR)

[Lange, F., Pfennigwerth, N., Hartl, R., Kerschner, H., Achleitner, D., Gatermann, S. G., et al. (2018). LMB-1, a novel family of class B3 MBLs from an isolate of Enterobacter cloacae. *Journal of Antimicrobial Chemotherapy* 73, 2331–2335. doi:](http://paperpile.com/b/whZSUZ/qQ8a) [10.1093/jac/dky215](http://dx.doi.org/10.1093/jac/dky215)[.](http://paperpile.com/b/whZSUZ/qQ8a)

[Lazzaro, L. M., Cassisi, M., Stefani, S., and Campanile, F. (2022). Impact of PBP4 Alterations on β-Lactam Resistance and Ceftobiprole Non-Susceptibility Among Enterococcus faecalis Clinical Isolates. *Frontiers in Cellular and Infection Microbiology* 11. doi:](http://paperpile.com/b/whZSUZ/dxWv) [10.3389/fcimb.2021.816657](http://dx.doi.org/10.3389/fcimb.2021.816657)[.](http://paperpile.com/b/whZSUZ/dxWv)

[Liakopoulos, A., Mevius, D., and Ceccarelli, D. (2016). A Review of SHV Extended-Spectrum β-Lactamases: Neglected Yet Ubiquitous. *Frontiers in Microbiology* 7. doi:](http://paperpile.com/b/whZSUZ/aTbD) [10.3389/fmicb.2016.01374](http://dx.doi.org/10.3389/fmicb.2016.01374)[.](http://paperpile.com/b/whZSUZ/aTbD)

[Livermore, D. M. (1995). beta-Lactamases in laboratory and clinical resistance. *Clinical Microbiology Reviews* 8, 557–584. doi:](http://paperpile.com/b/whZSUZ/tpKF) [10.1128/cmr.8.4.557](http://dx.doi.org/10.1128/cmr.8.4.557)[.](http://paperpile.com/b/whZSUZ/tpKF)

[Lowy, F. D. (2003). Antimicrobial resistance: the example of Staphylococcus aureus. *Journal of Clinical Investigation* 111, 1265–1273. doi:](http://paperpile.com/b/whZSUZ/ISKM) [10.1172/jci18535](http://dx.doi.org/10.1172/jci18535)[.](http://paperpile.com/b/whZSUZ/ISKM)

[Malachowa, N., and DeLeo, F. R. (2010). Mobile genetic elements of Staphylococcus aureus. *Cellular and Molecular Life Sciences* 67, 3057–3071. doi:](http://paperpile.com/b/whZSUZ/91gj) [10.1007/s00018-010-0389-4](http://dx.doi.org/10.1007/s00018-010-0389-4)[.](http://paperpile.com/b/whZSUZ/91gj)

[Ma, Z., Xu, C., Zhang, X., Wang, D., Pan, X., Liu, H., et al. (2021). A MexR Mutation Which Confers Aztreonam Resistance to Pseudomonas aeruginosa. *Frontiers in Microbiology* 12. doi:](http://paperpile.com/b/whZSUZ/lKor) [10.3389/fmicb.2021.659808](http://dx.doi.org/10.3389/fmicb.2021.659808)[.](http://paperpile.com/b/whZSUZ/lKor)

[Miller, W. R., Munita, J. M., and Arias, C. A. (2014). Mechanisms of antibiotic resistance in enterococci. *Expert Review of Anti-infective Therapy* 12, 1221–1236. doi:](http://paperpile.com/b/whZSUZ/ilK6) [10.1586/14787210.2014.956092](http://dx.doi.org/10.1586/14787210.2014.956092)[.](http://paperpile.com/b/whZSUZ/ilK6)

[Nordmann, P., Cuzon, G., and Naas, T. (2009). The real threat of Klebsiella pneumoniae carbapenemase-producing bacteria. *The Lancet Infectious Diseases* 9, 228–236. doi:](http://paperpile.com/b/whZSUZ/POJn) [10.1016/s1473-3099(09)70054-4](http://dx.doi.org/10.1016/s1473-3099(09)70054-4)[.](http://paperpile.com/b/whZSUZ/POJn)

[Ogbolu, D. O., Terry Alli, O. A., Webber, M. A., Oluremi, A. S., and Oloyede, O. M. (2018). CTX-M-15 is established in most multidrug-resistant uropathogenic Enterobacteriaceae and Pseudomonaceae from hospitals in Nigeria. *European Journal of Microbiology and Immunology* 8, 20–24. doi:](http://paperpile.com/b/whZSUZ/RAlR) [10.1556/1886.2017.00012](http://dx.doi.org/10.1556/1886.2017.00012)[.](http://paperpile.com/b/whZSUZ/RAlR)

[Ono, S., Muratani, T., and Matsumoto, T. (2005). Mechanisms of Resistance to Imipenem and Ampicillin in *Enterococcus faecalis*. *Antimicrobial Agents and Chemotherapy* 49, 2954–2958. doi:](http://paperpile.com/b/whZSUZ/kCEw) [10.1128/aac.49.7.2954-2958.2005](http://dx.doi.org/10.1128/aac.49.7.2954-2958.2005)[.](http://paperpile.com/b/whZSUZ/kCEw)

[Pai, H., Hong, J. Y., Byeon, J.-H., Kim, Y.-K., and Lee, H.-J. (2004). High Prevalence of Extended-Spectrum β-Lactamase-Producing Strains among Blood Isolates of *Enterobacter* spp. Collected in a Tertiary Hospital during an 8-Year Period and Their Antimicrobial Susceptibility Patterns. *Antimicrobial Agents and Chemotherapy* 48, 3159–3161. doi:](http://paperpile.com/b/whZSUZ/DLwI) [10.1128/aac.48.8.3159-3161.2004](http://dx.doi.org/10.1128/aac.48.8.3159-3161.2004)[.](http://paperpile.com/b/whZSUZ/DLwI)

[Paterson, G. K., Harrison, E. M., and Holmes, M. A. (2014). The emergence of mecC methicillin-resistant Staphylococcus aureus. *Trends in Microbiology* 22, 42–47. doi:](http://paperpile.com/b/whZSUZ/MqDC) [10.1016/j.tim.2013.11.003](http://dx.doi.org/10.1016/j.tim.2013.11.003)[.](http://paperpile.com/b/whZSUZ/MqDC)

[Philippon, A., Arlet, G., and Jacoby, G. A. (2002). Plasmid-Determined AmpC-Type β-Lactamases. *Antimicrobial Agents and Chemotherapy* 46, 1–11. doi:](http://paperpile.com/b/whZSUZ/kJ3l) [10.1128/aac.46.1.1-11.2002](http://dx.doi.org/10.1128/aac.46.1.1-11.2002)[.](http://paperpile.com/b/whZSUZ/kJ3l)

[Poulou, A., Voulgari, E., Vrioni, G., Koumaki, V., Xidopoulos, G., Chatzipantazi, V., et al. (2013). Outbreak Caused by an Ertapenem-Resistant, CTX-M-15-Producing Klebsiella pneumoniae Sequence Type 101 Clone Carrying an OmpK36 Porin Variant. *Journal of Clinical Microbiology* 51, 3176–3182. doi:](http://paperpile.com/b/whZSUZ/8YYg) [10.1128/jcm.01244-13](http://dx.doi.org/10.1128/jcm.01244-13)[.](http://paperpile.com/b/whZSUZ/8YYg)

[Rice, L. B., Carias, L. L., Hutton-Thomas, R., Sifaoui, F., Gutmann, L., and Rudin, S. D. (2001). Penicillin-Binding Protein 5 and Expression of Ampicillin Resistance in *Enterococcus faecium*. *Antimicrobial Agents and Chemotherapy* 45, 1480–1486. doi:](http://paperpile.com/b/whZSUZ/UJuH) [10.1128/aac.45.5.1480-1486.2001](http://dx.doi.org/10.1128/aac.45.5.1480-1486.2001)[.](http://paperpile.com/b/whZSUZ/UJuH)

[Rice, L. B., Desbonnet, C., Tait-Kamradt, A., Garcia-Solache, M., Lonks, J., Moon, T. M., et al. (2018). Structural and Regulatory Changes in PBP4 Trigger Decreased β-Lactam Susceptibility in Enterococcus faecalis. *mBio* 9. doi:](http://paperpile.com/b/whZSUZ/Kk7G) [10.1128/mbio.00361-18](http://dx.doi.org/10.1128/mbio.00361-18)[.](http://paperpile.com/b/whZSUZ/Kk7G)

[Salazar, M. J., Machado, H., Dillon, N. A., Tsunemoto, H., Szubin, R., Dahesh, S., et al. (2020). Genetic Determinants Enabling Medium-Dependent Adaptation to Nafcillin in Methicillin-Resistant Staphylococcus aureus. *mSystems* 5. doi:](http://paperpile.com/b/whZSUZ/yGL7) [10.1128/mSystems.00828-19](http://dx.doi.org/10.1128/mSystems.00828-19)[.](http://paperpile.com/b/whZSUZ/yGL7)

[Shaokat, S., Joly, B., Philippon, A., Sirot, D., and Cluzel, R. (1985). [Carbenicillin resistance of gram-negative bacteria: incidence, biochemical and genetic determinism]. *Pathol. Biol.*  33, 825–829.](http://paperpile.com/b/whZSUZ/xplJ)

[Signoretto, C., Boaretti, M., and Canepari, P. (1994). Cloning, sequencing and expression in*Escherichia coli*of the low-affinity penicillin binding protein of*Enterococcus faecalis*. *FEMS Microbiology Letters* 123, 99–106. doi:](http://paperpile.com/b/whZSUZ/3NxS) [10.1111/j.1574-6968.1994.tb07207.x](http://dx.doi.org/10.1111/j.1574-6968.1994.tb07207.x)[.](http://paperpile.com/b/whZSUZ/3NxS)

[Szabó, D., Melan, M. A., Hujer, A. M., Bonomo, R. A., Hujer, K. M., Bethel, C. R., et al. (2005). Molecular Analysis of the Simultaneous Production of Two SHV-Type Extended-Spectrum Beta-Lactamases in a Clinical Isolate of *Enterobacter cloacae* by Using Single-Nucleotide Polymorphism Genotyping. *Antimicrobial Agents and Chemotherapy* 49, 4716–4720. doi:](http://paperpile.com/b/whZSUZ/reLM) [10.1128/aac.49.11.4716-4720.2005](http://dx.doi.org/10.1128/aac.49.11.4716-4720.2005)[.](http://paperpile.com/b/whZSUZ/reLM)

[Verbist, L., and Verhaegen, J. (1986). Susceptibility of ticarcillin-resistant Gram-negative bacilli to different combinations of ticarcillin and clavulanic acid. *Journal of Antimicrobial Chemotherapy* 17, 7–15. doi:](http://paperpile.com/b/whZSUZ/PVEN) [10.1093/jac/17.suppl_c.7](http://dx.doi.org/10.1093/jac/17.suppl_c.7)[.](http://paperpile.com/b/whZSUZ/PVEN)

[Wachino, J.-I., Kurokawa, H., Suzuki, S., Yamane, K., Shibata, N., Kimura, K., et al. (2006). Horizontal Transfer of *bla* _CMY_ -Bearing Plasmids among Clinical *Escherichia coli* and *Klebsiella pneumoniae* Isolates and Emergence of Cefepime-Hydrolyzing CMY-19. *Antimicrobial Agents and Chemotherapy* 50, 534–541. doi:](http://paperpile.com/b/whZSUZ/nRiC) [10.1128/aac.50.2.534-541.2006](http://dx.doi.org/10.1128/aac.50.2.534-541.2006)[.](http://paperpile.com/b/whZSUZ/nRiC)

[Yan, J., Estanbouli, H., Liao, C., Kim, W., Monk, J. M., Rahman, R., et al. (2019). Systems-level analysis of NalD mutation, a recurrent driver of rapid drug resistance in acute Pseudomonas aeruginosa infection. *PLoS Comput. Biol.* 15, e1007562.](http://paperpile.com/b/whZSUZ/Gmqd)

[Zhang, X., Paganelli, F. L., Bierschenk, D., Kuipers, A., Bonten, M. J. M., Willems, R. J. L., et al. (2012). Genome-Wide Identification of Ampicillin Resistance Determinants in Enterococcus faecium. *PLoS Genetics* 8, e1002804. doi:](http://paperpile.com/b/whZSUZ/t0ht) [10.1371/journal.pgen.1002804](http://dx.doi.org/10.1371/journal.pgen.1002804)[.](http://paperpile.com/b/whZSUZ/t0ht)
